# EVA1A Regulates Hepatic Lipid Homeostasis by Modulating CD36 Expression and Its Palmitoylation

**DOI:** 10.34133/research.1001

**Published:** 2025-11-25

**Authors:** Di Yang, Lianhui Li, Kailai Zang, Wanyong Ma, Yuling Yang, Yani Sun, Bingqiang Zhang, Zunshuang Gong, Mingkang Yu, Qiyuan Du, Xiaokun Liu, Zhe Wang, Qiyue Xu, Ning Li

**Affiliations:** ^1^ Department of Biochemistry and Molecular Biology, School of Basic Medicine, Qingdao Medical College, Qingdao University, Qingdao, China.; ^2^ Emergency Department, Yantai Affiliated Hospital of Binzhou Medical University, Yantai, China.; ^3^ Department of Infectious Diseases, The Affiliated Hospital of Qingdao University, Qingdao University, Qingdao, China.; ^4^ Qingdao Restore Biotechnology Co., Ltd., Qingdao, China.; ^5^ Qingdao Engineering Research Center for Cellular Immunity and Early Cancer Screening, Qingdao, China.; ^6^ Department of Business, Qingdao University of Technology, Qingdao, China.

## Abstract

Hepatic lipid dysregulation drives metabolic dysfunction-associated steatotic liver disease (MASLD); nonetheless, the precise regulatory mechanisms remain incompletely elucidated. In this study, we examine the function of EVA1A, a known hepatocellular carcinoma tumor suppressor, in hepatic lipid metabolism. Hepatic EVA1A was markedly down-regulated in individuals diagnosed with MASLD, as well as in mice subjected to a high-fat diet. Hepatocyte-specific knockout of *Eva1a* in mice resulted in significant hepatic steatosis, accompanied by disrupted fatty acid metabolism, marked by increased fatty acid uptake and compromised β-oxidation, while hepatic Eva1a overexpression reversed these metabolic changes and largely alleviated fatty liver in ob/ob mice. Mechanistically, EVA1A deficiency activates mTORC1 (mechanistic target of rapamycin complex 1)–PPARγ2 (peroxisome proliferator-activated receptor γ2) signaling to up-regulate CD36 transcription. Concurrently, it transcriptionally represses the S-depalmitoylase APT1 while enhancing palmitoyl acyltransferases ZDHHC4/5, boosting CD36 palmitoylation. This dual action promotes CD36 plasma membrane localization for fatty acid uptake, reducing its mitochondrial distribution and impairing β-oxidation. Collectively, these results establish EVA1A as an essential regulator of hepatic lipid homeostasis, coordinating fatty acid uptake and β-oxidation by modulating CD36 expression and palmitoylation. Therefore, targeting the EVA1A–CD36 axis represents a promising therapeutic strategy for MASLD.

## Introduction

Metabolic dysfunction-associated steatotic liver disease (MASLD) has emerged as a pressing global health issue, with its rising prevalence closely paralleling the escalating rates of obesity and diabetes [1,2]. MASLD, marked by excessive accumulation of lipids in hepatocytes, is strongly linked to insulin resistance, inflammatory processes, and disruptions in hepatic glucose and lipid metabolism [3,4]. Fatty liver can lead to systemic metabolic dysfunction and evolve through a series of stages—from steatohepatitis and fibrosis to cirrhosis—ultimately culminating in hepatocellular carcinoma (HCC), an irreversible and life-threatening condition [5,6]. Currently, treatment options for MASLD are limited to lifestyle interventions, dietary modifications, and bariatric–metabolic surgery, and no effective drugs or pharmacotherapies have been approved for MASLD by the Food and Drug Administration [1], highlighting the critical necessity to identify therapeutic targets and feasible therapeutic strategies for MASLD. Therefore, gaining an understanding of the mechanisms that regulate hepatic lipid homeostasis is crucial for pinpointing potential intervention targets.

Hepatic lipid metabolic disorders caused by imbalances in lipid acquisition and utilization are the leading cause of MASLD. Fatty acid uptake is a vital physiological process in liver fat metabolism, facilitated by a soluble form of cluster of differentiation-36 (CD36) in coordination with the lipid-binding protein (FABP) and fatty acid transport protein (FATP) families [7,8]. CD36 is a class B scavenger receptor that shuttles dynamically between cell membrane and organelle membrane. It functions as both a lipid sensor and a regulator of lipid metabolism [9,10]. CD36 can be glycosylated, acetylated, phosphorylated, and palmitoylated, and these modifications strongly affect the binding ability of related ligands, thereby impacting various biological effects [10]. Among them, the dynamic palmitoylation of CD36 governs cellular fatty acid uptake by changing its location on the plasma membrane and endocytosis [11]. Numerous studies have shown that hepatic CD36 expression and its palmitoylation are significantly up-regulated in MASLD patients and animal models, thereby driving excessive fatty acid uptake and accelerating disease progression [12–14]. In mouse models, genetic deletion of CD36 mitigates high-fat diet (HFD)-induced hepatic steatosis [12]. Importantly, inhibiting CD36 palmitoylation pharmacologically redirects CD36 to mitochondria, facilitating fatty acid transport for β-oxidation and consequently improving MASLD phenotypes [13]. These findings collectively establish CD36 and its dynamic palmitoylation cycle as critical regulators of MASLD pathogenesis. However, the specific regulatory factors for CD36 expression and its palmitoylation within the liver remain unclear.

Eva-1 homolog A (EVA1A), also referred to as TMEM166 or FAM176A, is a protein associated with the endoplasmic reticulum and is predominantly expressed in liver. Recent studies have established EVA1A as a key player in several pathophysiological processes, including various cancers [15–20], Parkinson’s disease [21], embryonic neurogenesis [22], heart failure [23], acute liver injury [24], vascular endothelial repair [25], and atherosclerosis [26], by regulating autophagy and apoptosis. However, it is unknown whether it is involved in lipid metabolism. Our previous studies identified EVA1A, which is consistently down-regulated in the hepatic tissues of HCC patients, as a tumor suppressor in HCC [7,8]. Pathology data indicated that HCC patients with down-regulated EVA1A were mostly accompanied by hepatic steatosis [7]. Another study showed that CCAAT/enhancer binding protein alpha is up-regulated in a portion of HCC patients and may promote lipid decomposition through EVA1A, providing energy for tumor cell survival [27]. Based on these findings and the high hepatic expression of Eva1a, we hypothesize that EVA1A could contribute to hepatic lipid metabolism; however, its exact function in this process is completely unknown.

The present investigation aimed to explore the role of EVA1A in the modulation of hepatic lipid homeostasis and the pathogenesis of MASLD. Our findings revealed a significant down-regulation of hepatic EVA1A expression in patients with MASLD and in mice subjected to an HFD. Liver-specific *Eva1a* knockout in healthy mice directly induced hepatic steatosis; however, hepatic *Eva1a* overexpression in ob/ob mice largely alleviated the hepatic steatosis, demonstrating EVA1A’s protective role in liver lipid metabolism. Using genetic manipulation in primary rat hepatocytes and HepG2/Huh7 cell lines, we discovered that EVA1A modulates hepatic lipid metabolism by suppressing fatty acid uptake and promoting β-oxidation. CD36 was identified as a crucial downstream effector of EVA1A. EVA1A governs CD36 function through a dual regulatory mechanism: (a) transcriptionally, by suppressing CD36 expression via the mTORC1 (mechanistic target of rapamycin complex 1)–PPARγ2 (peroxisome proliferator-activated receptor γ2) axis; and (b) posttranslationally, by inhibiting CD36 palmitoylation, thereby modulating its translocation between the plasma membrane and mitochondria. Here, we established for the first time EVA1A as a critical modulator of hepatic lipid metabolism, whose down-regulation promotes the initiation and progression of MASLD. These findings provide evidence that targeting the EVA1A–CD36 axis represents a novel potential strategy for MASLD treatment.

## Results

### Hepatic EVA1A expression is reduced in MASLD patients and animal models

**Fig. 1.**
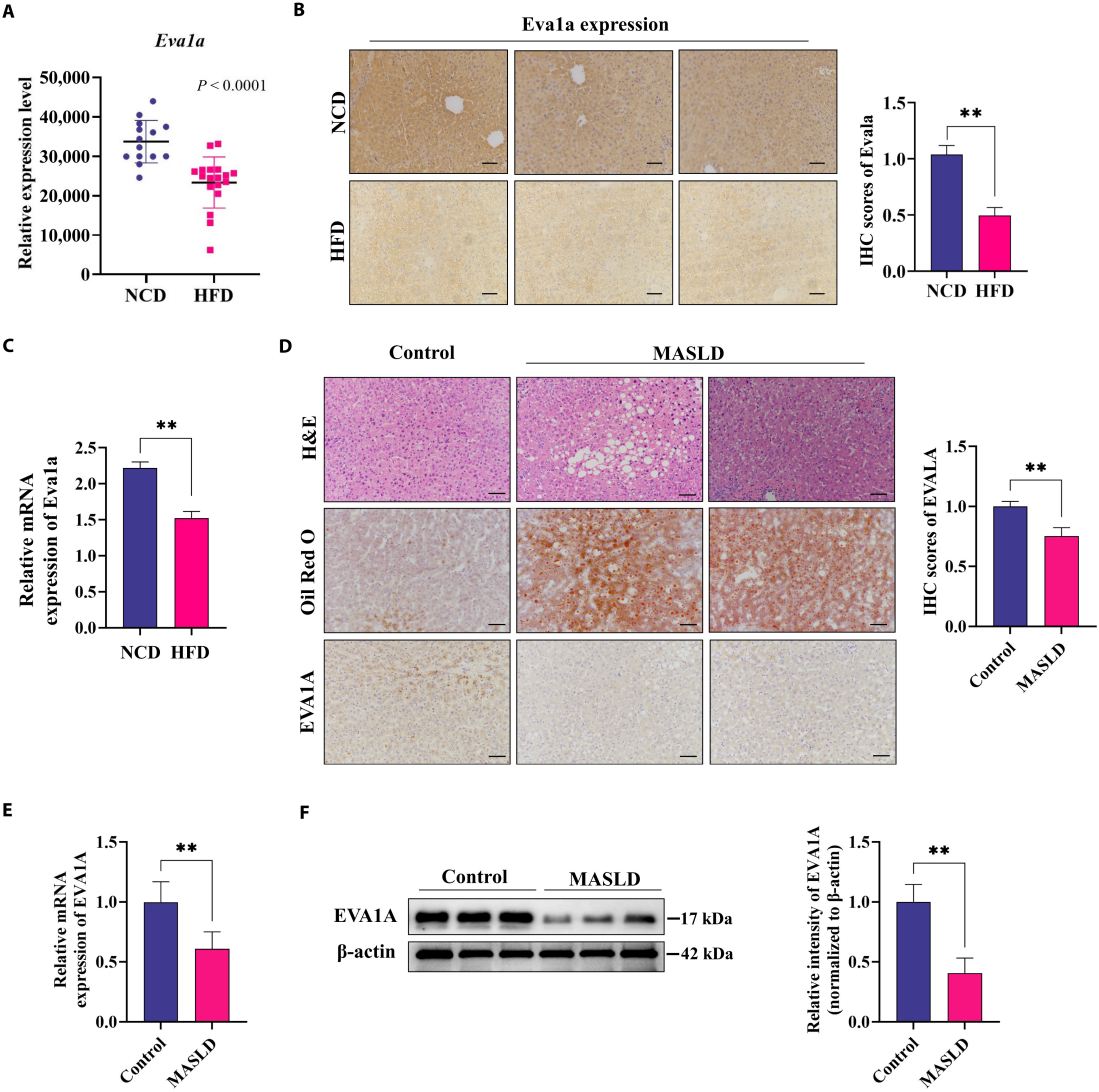
In fatty livers, hepatic EVA1A expression is reduced. (A) Scatter diagram illustrating the relative liver *Eva1a* transcripts in the HFD group in comparison to the NCD controls, based on microarray data obtained from the GEO database. (B) Immunohistochemical (IHC) analysis of hepatic Eva1a expression in HFD-induced MASLD mice and NCD-fed mice. IHC scores of Eva1a were quantified in the right panels (*n* = 5). Scale bars: 100 μm. (C) RT-qPCR analysis was performed to determine the relative hepatic *Eva1a* mRNA levels in HFD-induced MASLD mice compared to the NCD controls (*n* = 5). (D) Representative H&E, Oil Red O, and IHC staining for EVA1A in liver sections from MASLD and control individuals (*n* = 8). Scale bars: 100 μm. IHC scores of EVA1A were quantified in the right panels. (E) Analysis of relative hepatic *EVA1A* mRNA expression by RT-qPCR in MASLD individuals and controls (*n* = 8). (F) Hepatic EVA1A expression was assessed by Western blot in patients with MASLD and controls (*n* = 3). β-actin served as a loading control. **P < 0.01.

To elucidate the possible function of EVA1A in MASLD pathogenesis, we first analyzed a published dataset from the Gene Expression Omnibus (GEO) database, generated from liver tissues of HFD-induced MASLD mice (https://www.ncbi.nlm.nih.gov/geo/query/acc.cgi?acc=GSE83596). Our analysis revealed a significantly lower transcript level of EVA1A in the livers of HFD-fed mice compared with those fed a normal chow diet (NCD) (Fig. 1A). In our previous HFD-induced MASLD mouse model, the protein levels and transcript levels of hepatic Eva1a were confirmed to be significantly down-regulated compared with those in NCD-fed mice (Fig. 1B and C). Moreover, we also observed that both the protein expression level and the transcript levels of hepatic EVA1A were markedly down-regulated in patients with MASLD, as revealed by immunohistochemical (IHC) staining, RT-qPCR (real-time quantitative polymerase chain reaction) analysis, and Western blot analysis (Fig. 1D to F). These findings suggest that hepatic EVA1A level is notably reduced in MASLD.

### Liver-specific deletion of Eva1a induces hepatic steatosis in mice

**Fig. 2.**
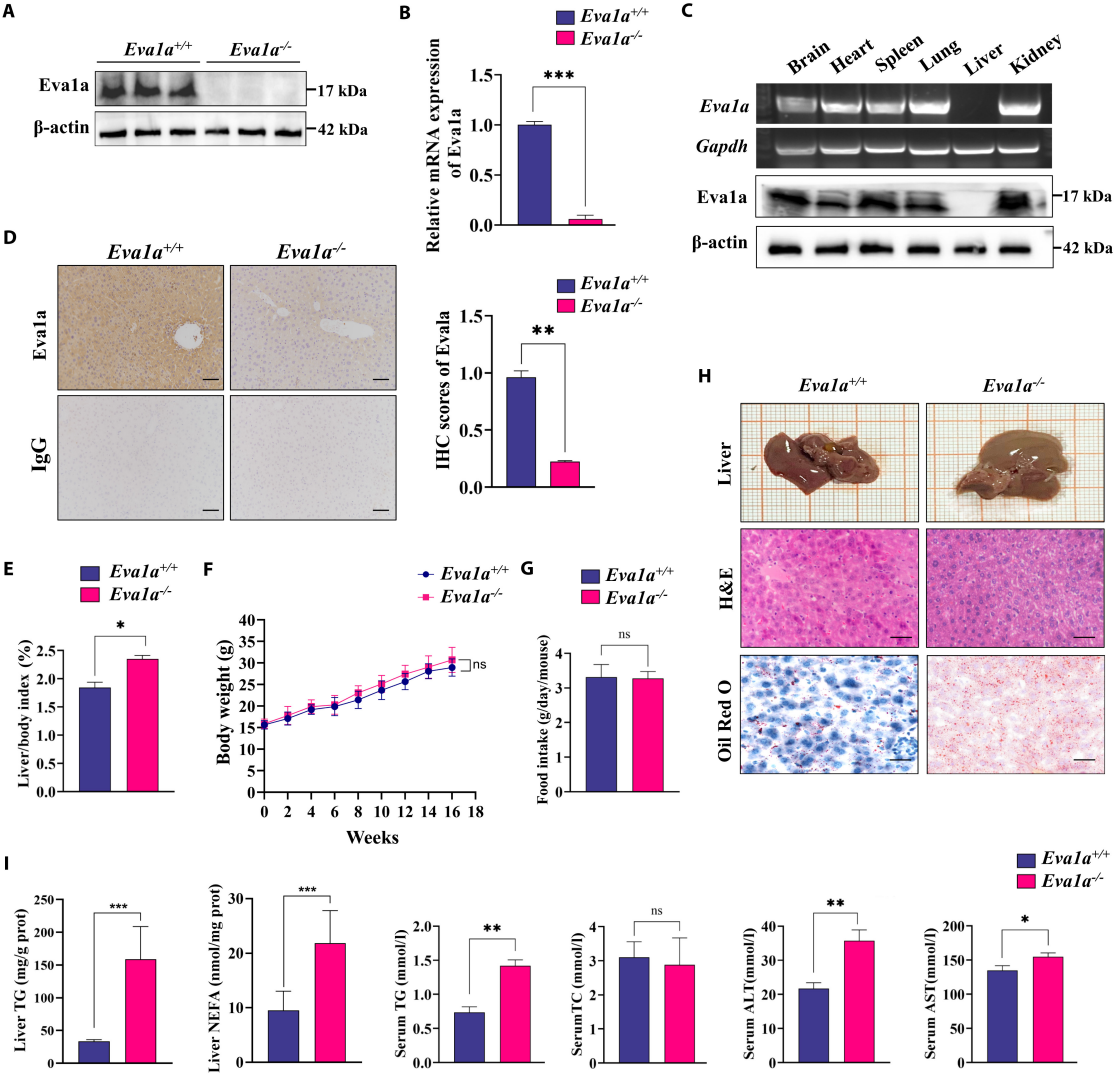
*Eva1a* knockout induces hepatic steatosis in mice. (A) Successful deletion of EVA1A protein in hepatic tissue was confirmed through Western blotting (*n* = 3). β-actin served as a loading control. (B) RT-qPCR analysis was performed to determine the relative *Eva1a* mRNA levels in liver of *Eva1a^+/+^*mice and *Eva1a^−/−^* mice (*n* = 8). (C) The representative genomic DNA PCR results and Western blot results showing the levels of exon 3 of *Eva1a* and the expression of Eva1a protein in different tissues of *Eva1a^−/−^* mice (*n* = 3). (D) Representative IHC staining of Eva1a in *Eva1a^+/+^* and *Eva1a^−/−^* mice liver tissues (*n* = 4). The negative control was stained by isotype IgG. The right panels show the Eva1a IHC quantification. Scale bars: 100 μm. (E) The liver index (liver-to-body weight ratios) (*n* = 8). (F) The body weight of *Eva1a^+/+^* and *Eva1a^−/−^* mice during the experiment (*n* = 8). (G) Average daily food intake during the experiment (*n* = 8). (H) Representative photomicrographs of liver histology: gross morphology, H&E, and Oil Red O staining (*n* = 4). Scale bars: 50 μm. (I) Liver TG and NEFA levels (*n* = 4). Serum TG, TC, ALT, and AST levels (*n* = 8). *P < 0.05, **P < 0.01, ***P < 0.001. ns means no significance.

To explore the biological function of EVA1A in hepatic tissue, we created liver-specific Eva1a knockout (*Eva1a*-LKO) mice (*Eva1a^−/−^* mice) using the Cre/loxP recombination system (Fig. S1A to C). *Eva1a^−/−^* mice were identified via Western blot and RT-qPCR analyses of liver tissues. The results demonstrated that the protein and mRNA levels of Eva1a were significantly decreased (Fig. 2A and B). In addition, PCR and Western blot analysis revealed that the *Eva1a* gene was specifically knocked out in the livers of the mice, with minimal changes observed in the brain, heart, spleen, lung, or kidney tissues (Fig. 2C). To further evaluate Eva1a protein expression, we performed IHC staining on liver tissue from *Eva1a*^+/+^ and *Eva1a^−/−^* mice, which confirmed the knockout effect (Fig. 2D). By comparing *Eva1a^+/+^* and *Eva1a^−/−^* mice, we observed that the liver index of *Eva1a^−/−^* mice increased significantly (Fig. 2E), while body weight and food intake did not differ significantly between groups throughout the experiment (Fig. 2F and G). *Eva1a^−/−^* mice exhibited enlarged livers that appeared yellow in color. Liver histology and Oil Red O (ORO) staining demonstrated that *Eva1a^−/−^* mice developed significant hepatic steatosis (Fig. 2H). Furthermore, the triglyceride (TG) levels and nonesterified fatty acid (NEFA) levels in the liver, serum TG levels, alanine aminotransferase (ALT) levels, and aspartate aminotransferase (AST) levels were notably increased in *Eva1a^−/−^* mice (Fig. 2I). Collectively, these findings demonstrate that targeted deletion of Eva1a in the liver leads to the development of hepatic steatosis in mice.

### Hepatic EVA1A deficiency enhances the expression of genes implicated in fatty acid uptake

**Fig. 3.**
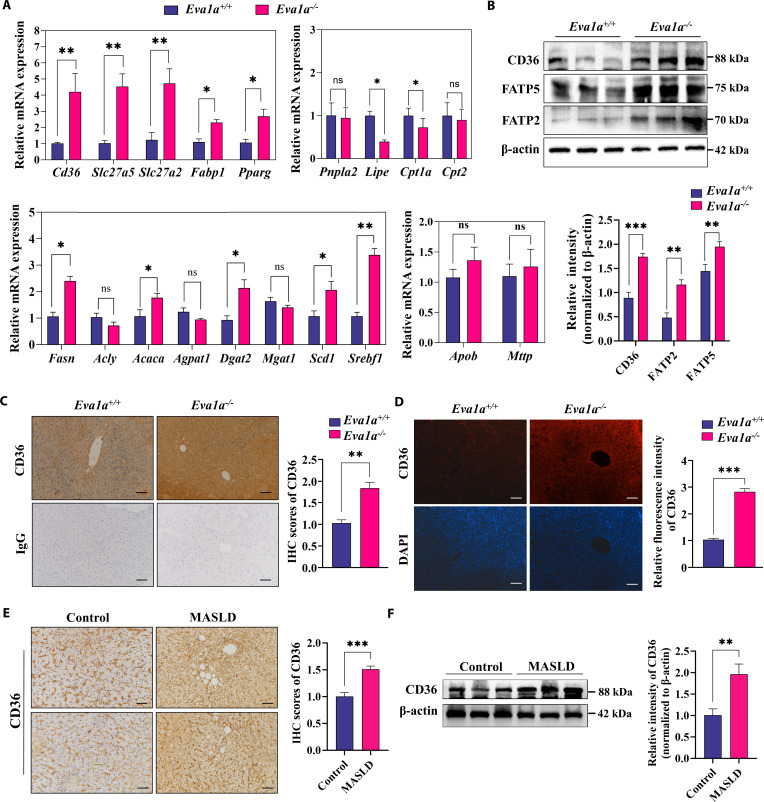
*Eva1a* knockout enhances the expression of genes implicated in fatty acid uptake. (A) RT-qPCR analysis of the relative mRNA levels of genes associated with fatty acid transport (*Cd36*, *Slc27a5*, *Slc27a2*, and *Fabp1*) and related transcription factor *Pparg2*, lipid synthesis genes (*Fasn*, *Acly*, *Acaca*, *Agpat1*, *Dgat2*, *Mgat1*, and *Scd1*) and related transcription factor (*Srebf1*), lipolysis and β-oxidation genes (*Pnpla2* [encoding ATGL], *Lipe*, *Cpt1a*, and *Cpt2*), and lipid assembly and secretion genes (*Apob* and *Mttp*) in liver tissues of *Eva1a^+/+^* mice and *Eva1a^−/−^* mice (*n* = 4). (B) Western blot analysis of fatty acid transport protein CD36, FATP5, and FATP2 in mice liver tissues (*n* = 3). The blot bands were quantified by normalization to β actin by ImageJ in the lower panels. (C) Representative IHC staining image of CD36 in liver sections from mice. Scale bars: 100 μm. IHC scores of CD36 were quantified from 4 mice in the right panels. (D) Representative immunofluorescence staining image of CD36 protein (red) and nucleus (stained with DAPI, blue) in mice liver tissues (*n* = 4). Scale bars: 100 μm. The right panels show the CD36 fluorescence intensity quantified using ImageJ software. (E) Comparative representative images of CD36 IHC staining in liver sections from MASLD patients and control subjects (*n* = 4). Scale bars: 100 μm. IHC scores of CD36 were quantified in the right panels (*n* = 4). (F) The hepatic CD36 expression from MASLD individuals and controls were analyzed by Western blot (*n* = 3). *P < 0.05, **P < 0.01, ***P < 0.001.

Based on the observations that *Eva1a* knockout triggered hepatic steatosis in mice, we postulated that EVA1A plays a protective role against MASLD. To investigate the mechanism by which hepatic EVA1A regulates lipid deposition in vivo, we therefore quantified the mRNA levels of key enzymes across major lipid metabolic pathways. In the livers of *Eva1a^−/−^* mice, both the transcripts of fatty acid transport proteins (*Cd36*, *Slc27a2*, and *Slc27a5*) and the enzymes involved in fat synthesis (*Fasn, Acaca, Dgat2*, and *Scd1*), as well as the related transcription factors (*Pparg2* and *Srebf1*), were significantly increased, whereas the transcripts of the key enzymes involved in lipolysis (*Lipe*, encoding HSL) and fatty acid β-oxidation (*Cpt1a*) were significantly reduced (Fig. 3A). Compared with *Eva1a^+/+^* mice, *Eva1a^−/−^* mice presented no significant alterations in the expression of genes associated with lipid assembly or secretion, including Apob and Mttp (Fig. 3A). We subsequently investigated the impact of Eva1a on protein expression associated with fatty acid uptake. Western blot analysis demonstrated a marked up-regulation in the protein levels of CD36, FATP5 (encoded by Slc27a5), and FATP2 (encoded by Slc27a2) in the liver tissues of Eva*1a^−/−^* mice (Fig. 3B). Consistently, IHC and immunofluorescence staining of liver tissue revealed a notable increase in Cd36 expression in *Eva1a^−/−^* mice (Fig. 3C and D). Similarly, the expression of CD36 in liver tissues from MASLD patients showed a marked elevation, as demonstrated by IHC staining and Western blot analysis (Fig. 3E and F). Collectively, these data indicate that EVA1A may play a role in hepatic lipid metabolism, particularly in the process of fatty acid uptake.

### EVA1A negatively regulates fatty acid uptake and lipid accumulation in hepatocytes

**Fig. 4.**
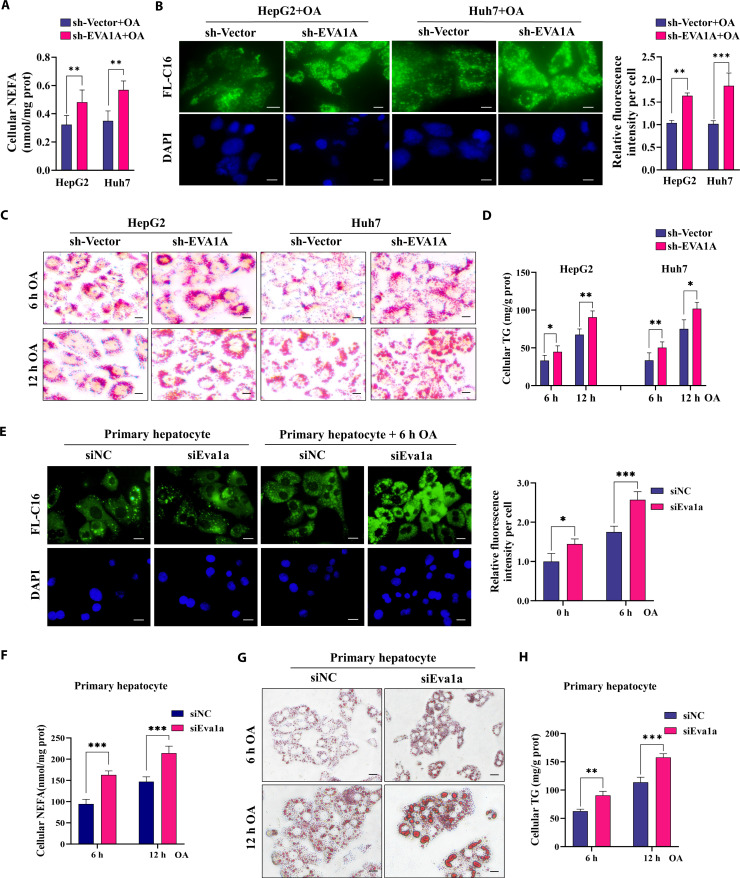
EVA1A knockdown enhances fatty acid uptake and increases lipid droplet accumulation. (A) The cellular NEFA levels in EVA1A-knockdown HepG2 and Huh7 cells, along with control cells, treated with 400 μM OA for 6 h, were quantified and the values were means ± SDs (*n* = 3). (B) Representative fluorescence images of cellular fatty acids (stained with BODIPY FL-C16, green) and nucleus (stained with DAPI, blue) in cells treated with 400 μM OA for 6 h. Scale bars: 5 μm. The fluorescence signal intensity of FL-C16 per cell was quantified by ImageJ, *n* = 50 cells. (C) Representative microscopy images of ORO staining for lipid droplets after 400 μM OA treatment for 6 or 12 h in HepG2 and Huh7 cells. Scale bars: 5 μm. (D) The cellular TG contents in HepG2 and Huh7 cells. (E) Representative fluorescence images of BODIPY FL-C16 staining for internalized fatty acids in EVA1A-knockdown primary rat hepatocytes, along with control cells, after a 6-h treatment with 400 μM OA or left untreated. Scale bars: 5 μm. The fluorescence signal intensity of FL-C16 per cell was quantified by ImageJ in the right panels. Values were means ± SDs, *n* = 50. (F) The cellular NEFA levels in primary rat hepatocytes. (G) Representative microscopy images of ORO staining for lipid droplets in primary rat hepatocytes treated with 400 μM OA for 6 or 12 h. Scale bars: 10 μm. (H) The cellular TG contents in primary rat hepatocytes. *P < 0.05, **P < 0.01, ***P < 0.001. RFU, relative fluorescence units.

To further confirm the function of EVA1A in fatty acid uptake, we established stable EVA1A-knockdown and EVA1A-overexpressing cell lines in HepG2 and Huh7 cells by transfecting them with an shRNA plasmid targeting EVA1A or LV-EVA1A lentivirus, respectively. We assessed the stable expression of EVA1A using a combination of fluorescence imaging, RT-qPCR, and Western blot analysis (Fig. S2A to C). These 2 cell lines were treated with oleic acid (OA) to mimic lipid overload conditions. A Cell Counting Kit-8 (CCK-8) assay revealed that treatment with an OA concentration below 0.5 mmol/l for less than 12 h had little effect on hepatocyte viability (Fig. S3). To dissect the function of EVA1A in fatty acid uptake, we measured the cellular NEFA levels and stained the internalized fatty acids with the fluorescent dye BODIPY FL-C16. EVA1A knockdown resulted in significantly higher levels of cellular NEFAs and internalized fatty acids in 400 μM OA-treated HepG2 and Huh7 cells (Fig. 4A and B), even in the absence of OA exposure (Fig. S4A and B). In addition, the intracellular lipid droplets synthesized from ingested fatty acids or synthesized internally were stained with ORO or BODIPY 493/503. Upon stimulation with 400 μM OA, a time-dependent accumulation of these droplets was observable in EVA1A-knockdown cells, with a concomitant rise in cellular TG content (Fig. 4C, Fig. S5A, and Fig. 4D). Notably, even without OA exposure, EVA1A knockdown resulted in significant accumulation of intracellular lipid droplets (Fig. S5A). To further confirm these results, we isolated primary rat hepatocytes and knocked down the expression of Eva1a in them with siRNA (Fig. S6). We consistently observed that the knockdown of Eva1a led to a significant increase in internalized fatty acids and a notable elevation of cellular NEFA levels in primary rat hepatocytes, both at baseline and following OA exposure (Fig. 4E and F). Additionally, the accumulation of intracellular lipid droplets and TG levels increased significantly in a time-dependent manner (Fig. 4G and H).

**Fig. 5.**
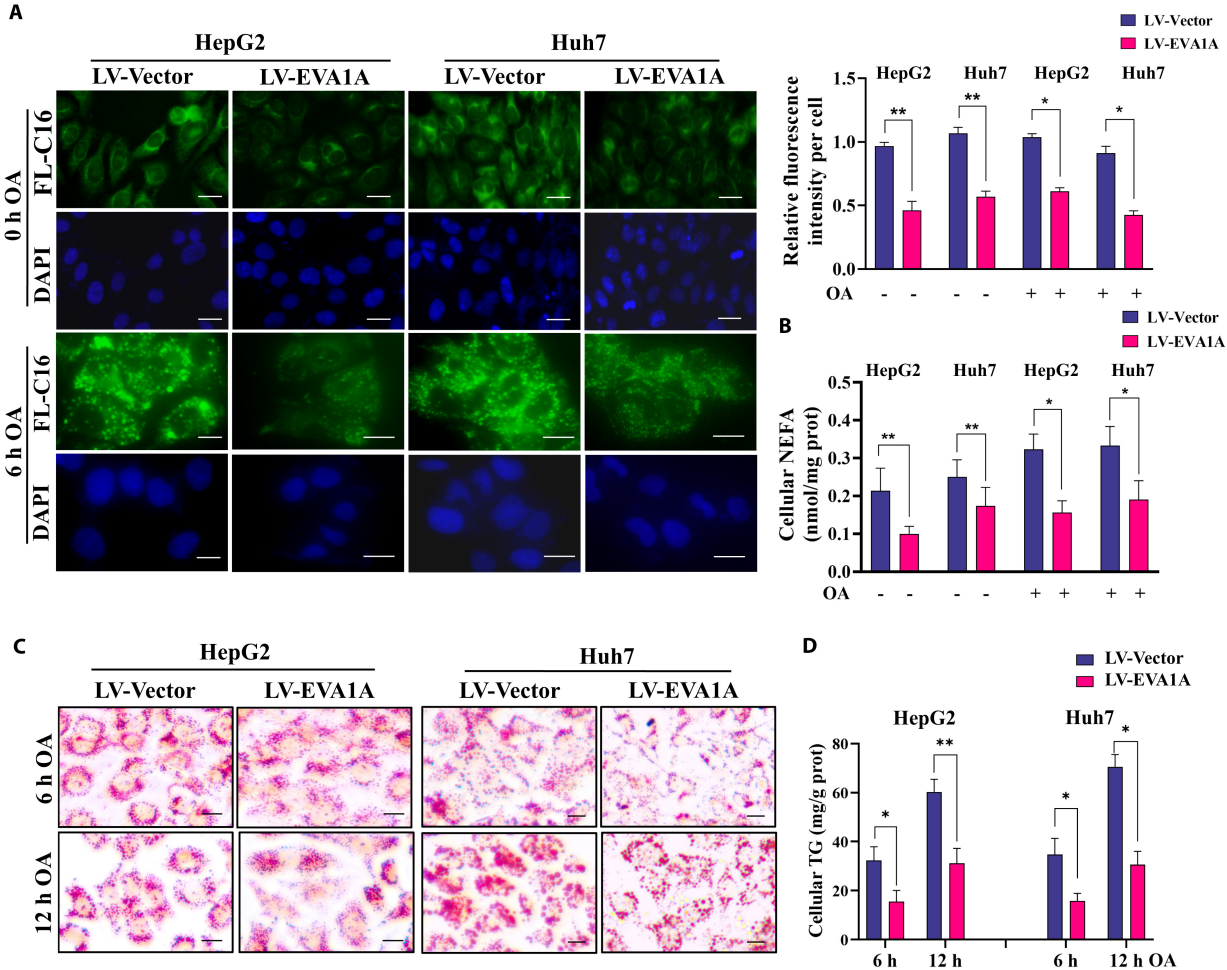
EVA1A overexpression reduces fatty acid uptake and decreases lipid droplet accumulation. (A) Representative fluorescence images of internalized fatty acids in control cells or EVA1A-overexpressed HepG2 and Huh7 cells after a 6-h treatment with 400 μM OA or left untreated. Scale bars: 10 μm. The fluorescence signal intensity of FL-C16 per cell was quantified by ImageJ in the right panels, *n* = 50 cells. (B) The cellular NEFA levels in control cells or EVA1A-overexpressed HepG2 and Huh7 cells after a 6-h treatment with 400 μM OA or after being left untreated. (C) Representative microscopy images of ORO staining for lipid droplets in control cells or EVA1A overexpressed HepG2 and Huh7 cells treated with 400 μM OA for 6 or 12 h. Scale bars: 10 μm. (D) The cellular TG contents were determined. *P < 0.05, **P < 0.01.

Conversely, EVA1A overexpression led to a significant reduction in internalized fatty acids and cellular NEFA levels in HepG2 and Huh7 cells (Fig. 5A and B), as well as a notable decrease in lipid droplet accumulation and cellular TG levels (Fig. 5C, Fig. S5B, and Fig. 5D) following OA exposure or not. These findings suggest that EVA1A negatively regulates fatty acid uptake both under pathological conditions and in the context of lipid overload. Collectively, these data indicate that EVA1A inhibits hepatic fatty acid uptake and reduces hepatic lipid droplet formation.

### CD36 mediates EVA1A deletion-induced enhancement of fatty acid uptake and lipid accumulation

**Fig. 6.**
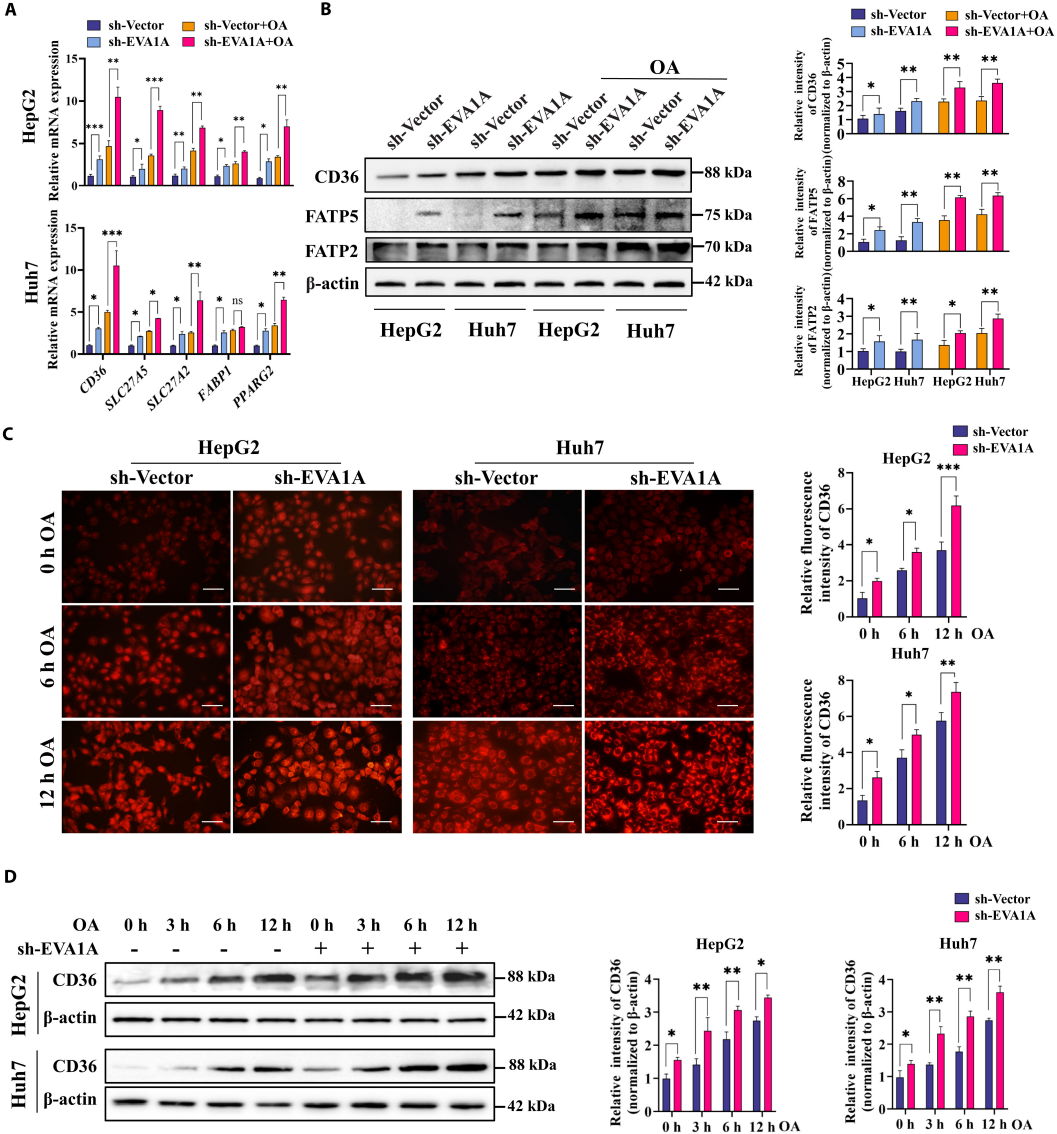
EVA1A knockdown up-regulates the expression of genes involved in fatty acid transport. (A) RT-qPCR analysis of the relative mRNA levels of genes associated with fatty acid intake (*CD36*, *SLC27A5*, *SLC27A2*, and *FABP1*) and related transcription factor *PPARG2* in EVA1A-knockdown HepG2 and Huh7 cells, along with the control cells, after a 6-h treatment with 400 μM OA or left untreated. (B) Cells were treated following the protocol in panel (A) and then subjected to Western blot analysis to detect the proteins associated with fatty acid intake (CD36, FATP5, and FATP2). Protein levels were quantified in the right panels. (C) Following treatment with either 400 μM OA for 6 or 12 h or no treatment, EVA1A-knockdown cells or control cells were fixed, stained with CD36 antibody and imaged by fluorescence microscopy. Scale bars: 50 μm. Quantification of CD36 fluorescence intensity (right panels) was performed with ImageJ. (D) Assessment of CD36 expression by Western blot in EVA1A-knockdown cells or control cells under the treatment with OA for 0, 3, 6, and 12 h. Protein levels of CD36 were quantified in the right panels. *P < 0.05, **P < 0.01, ***P < 0.001.

**Fig. 7.**
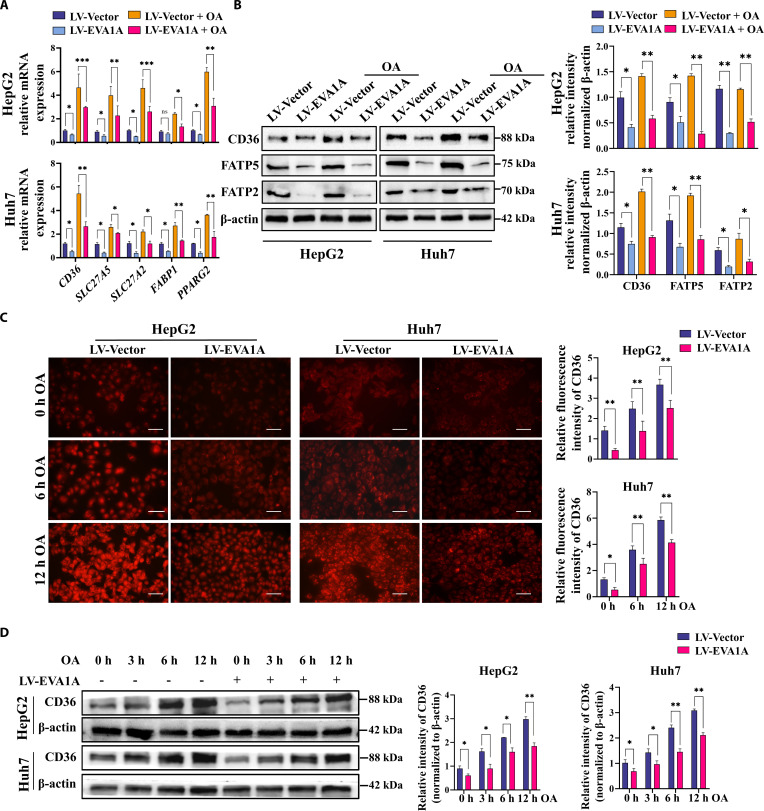
EVA1A overexpression inhibits the expression of genes involved in fatty acid transport. (A) RT-qPCR analysis of the relative mRNA levels of fatty acid uptake genes (*CD36*, *SLC27A5*, *SLC27A2*, and *FABP1*) and related transcription factor *PPARG2* in EVA1A-overexpressed HepG2 and Huh7 cells, along with control cells, after a 6-h treatment with 400 μM OA or left untreated. (B) Following the scheme in panel (A), cells were treated and then subjected to Western blot analysis to detect the proteins (CD36, FATP5, and FATP2). Protein levels were quantified in the right panels. (C) After 6- or 12-h treatment with 400 μM OA or left untreated, EVA1A-overexpressed cells or control cells were fixed, stained with CD36 antibody, and imaged by fluorescence microscopy. Scale bars: 50 μm. CD36 fluorescence intensity was measured with ImageJ in the right panels. (D) Western blot analysis of CD36 expression in EVA1A-overexpressing and control cells treated with 400 μM OA for the indicated times (0, 3, 6, and 12 h). Protein levels of CD36 were quantified in the right panels. *P < 0.05, **P < 0.01, ***P < 0.001.

To elucidate the possible mechanism by which EVA1A regulates fatty acid uptake, we assessed the expression of fatty acid transport proteins and the correlated transcription factor PPARγ2 in HepG2 and Huh7 cells. Similar to the findings in *Eva1a -*LKO mice, EVA1A knockdown resulted in a significant up-regulation of *CD36, SLC27A2, SLC27A5, FABP1*, and *PPARG2* (encoding PPARγ2) mRNAs in HepG2 and Huh7 cells, even with OA exposure (Fig. 6A) and a marked up-regulation of *CD36, SLC27A2,* and *SLC27A5* mRNAs in primary rat hepatocytes (Fig. S6). Consistently, under the same conditions, Western blot results confirmed that the protein levels of CD36, FATP5, and FATP2 were markedly elevated (Fig. 6B). Furthermore, immunofluorescence staining and Western blot analysis revealed that CD36 expression progressively increased with prolonged OA treatment duration, and EVA1A knockdown further amplified this up-regulation (Fig. 6C and D). However, in EVA1A-overexpressing HepG2 or Huh7 cells, the mRNA levels of *CD36*, *SLC27A5*, *SLC27A2*, *FABP1*, and *PPARG2*, as well as the expression of CD36, FATP5, and FATP2, were markedly reduced under the same conditions (Fig. 7A and B). Moreover, EVA1A overexpression significantly attenuated the OA-induced up-regulation of CD36 expression across different treatment durations (Fig. 7C and D). Collectively, these findings demonstrate that EVA1A may negatively regulate the fatty acid uptake and transport gene expression.

**Fig. 8.**
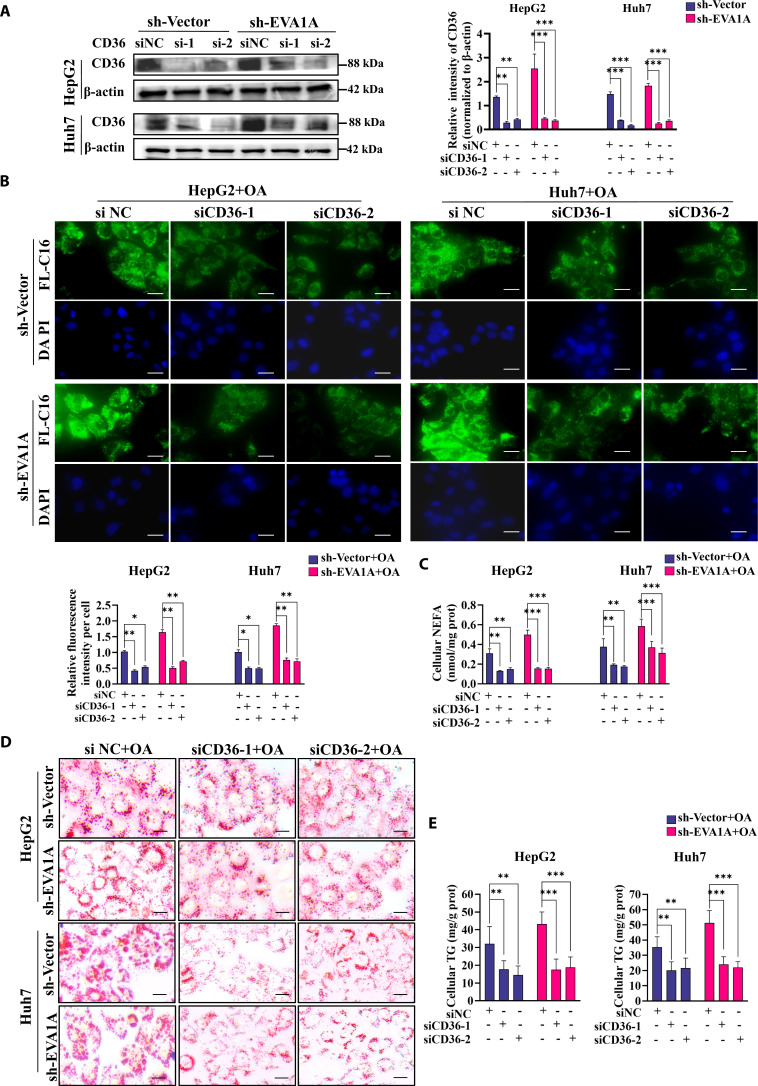
CD36 mediates the enhancement of fatty acid uptake and lipid droplet accumulation induced by EVA1A deletion. (A) EVA1A-knockdown HepG2 and Huh7 cells, along with control cells, were transfected with siNC or siCD36-1 or siCD36-2; 48 h later, cell lysates were collected and the knockdown efficiency of CD36 was assessed by Western blot. Protein levels of CD36 were quantified in the lower panels. (B) Cells were treated as (A) indicated, finally exposed to 400 μM OA for the last 6 h, then were fixed, stained with BODIPY FL-C16 (labeled fatty acids) and DAPI (labeled nucleus), and imaged by fluorescence microscopy. Scale bars: 20 μm. The fluorescence signal intensity of FL-C16 per cell was quantified by ImageJ in the lower panels (*n* = 50). (C) NEFA levels in cells treated as (B) indicated. (D) Representative images of ORO staining for lipid droplets in cells treated according to the scheme in panel (B). Scale bars: 10 μm. (E) Quantitative results of cellular TG contents. *P < 0.05, **P < 0.01, ***P < 0.001.

Given that CD36 serves as the primary receptor responsible for the initial interaction with fatty acids, we then knocked down CD36 in EVA1A-deficient HepG2 and Huh7 cells with 2 separate siRNAs and assessed the changes in fatty acid uptake and lipid droplet accumulation. Western blot and RT-qPCR analyses were performed to confirm the down-regulation of CD36 expression (Fig. 8A and Fig. S7A). BODIPY FL-C16 staining and cellular NEFA measurements demonstrated that CD36 ablation completely rescued the EVA1A deletion-induced fatty acid internalization after treatment with OA or left untreated (Fig. 8B and C and Fig. S7B and C). Consistently, ORO staining demonstrated that CD36 knockdown significantly reduced lipid droplet accumulation caused by EVA1A deletion during OA exposure (Fig. 8D), which coincided with a substantial reduction in the cellular TG content (Fig. 8E). These findings suggest that EVA1A deficiency relies on up-regulation of CD36 to promote fatty acid uptake and lipid droplet accumulation.

### EVA1A deficiency facilitates CD36 plasma membrane localization

**Fig. 9.**
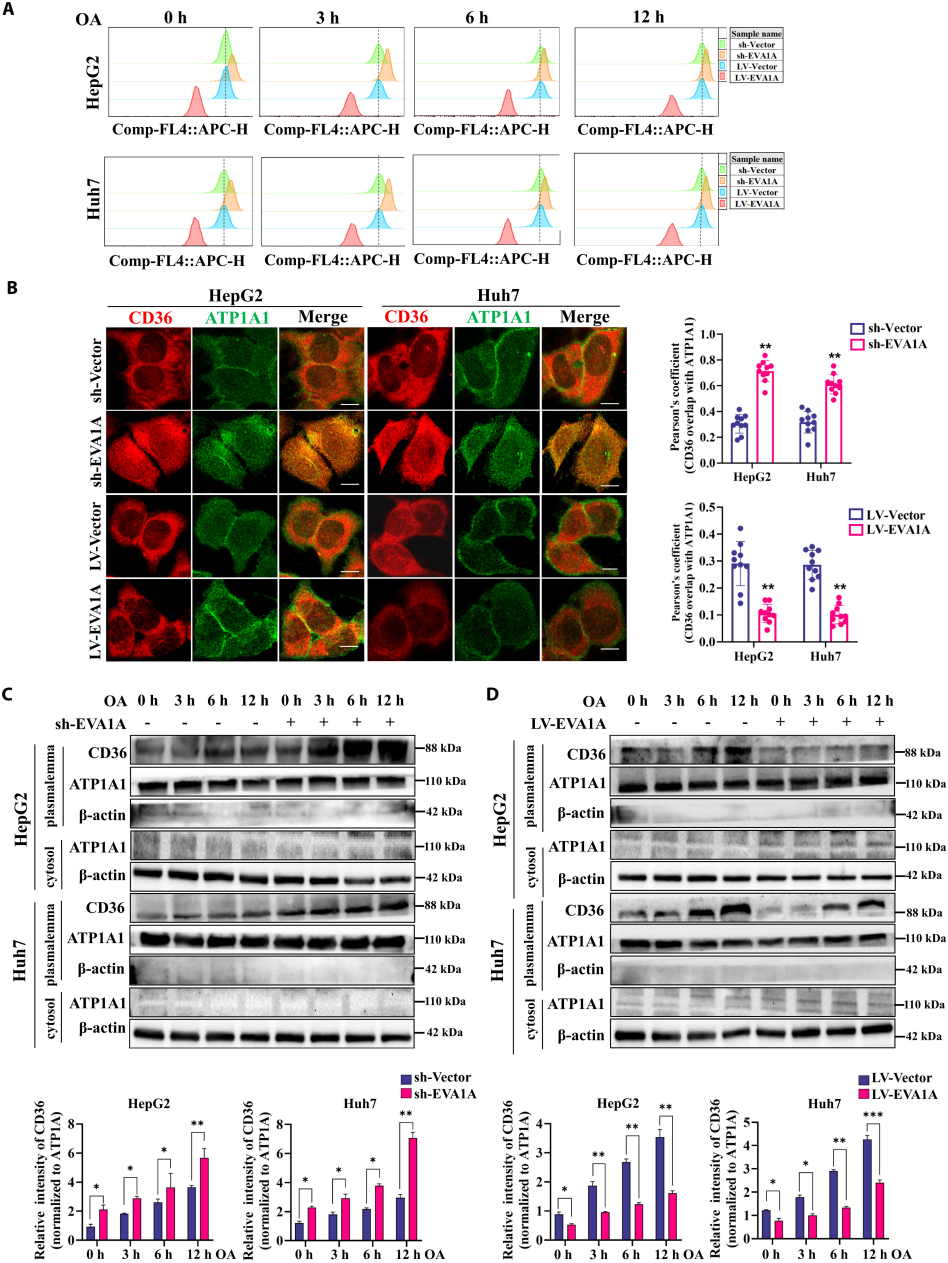
EVA1A knockdown promotes the plasma membrane localization of CD36. (A) HepG2 and Huh7 cells with EVA1A knockdown or overexpression, along with their corresponding control cells, were treated with 400 μM OA for 0, 3, 6, and 12 h, then the cell surface CD36 was labeled with allophycocyanin (APC)-conjugated CD36 antibody and detected by flow cytometry. The further the peak of the fluorescence signal shifts to the right, the more CD36 is present on the cell membrane. (B) EVA1A knockdown or overexpression cells, along with their corresponding control cells, were subjected to confocal microscopy analysis after staining with CD36 (red) and ATP1A1 (green) antibodies. Scale bars: 5 μm. Colocalization analysis is shown in the right panel (*n* = 10). (C and D) The 2 types of cells were treated as (A) indicated, then were collected, the plasma membrane fractions and cytosol fractions were extracted, and analyzed by Western blot to assess the expression of CD36, ATP1A1, and β-actin. Cell membrane CD36 levels were quantified in the lower panels. *P < 0.05, **P < 0.01, ***P < 0.001.

Given that CD36 binds fatty acids on the plasma membrane, we next sought to determine whether EVA1A regulates the subcellular localization of CD36 to the plasma membrane. To do so, we labeled cell surface CD36 in HepG2 and Huh7 cells using an allophycocyanin (APC)-conjugated CD36 antibody. Flow cytometry analysis revealed that EVA1A knockdown increased the cell membrane surface CD36 levels, whereas EVA1A overexpression decreased the cell membrane surface CD36 levels under varying conditions of 0 to 12 h of treatment with 400 μM OA (Fig. 9A). To better illustrate the influence of EVA1A on the cell membrane localization of CD36, we investigated the colocalization of CD36 with ATP1A1, a plasma membrane marker, via laser confocal microscopy. As shown in Fig. 9B, EVA1A-knockdown HepG2 and Huh7 cells exhibited enhanced colocalization of CD36 and ATP1A1 signals compared to control cells, whereas EVA1A-overexpressing cells showed reduced colocalization. Subsequently, plasma membrane fractions were isolated to evaluate the influence of EVA1A deficiency on the plasma membrane localization of CD36. As illustrated in Fig. 9C and D, with prolonged OA exposure time, the level of the CD36 protein on the plasma membrane gradually increased, while EVA1A knockdown further amplified its distribution on the plasma membrane. Conversely, EVA1A overexpression markedly reduced its expression on the plasma membrane. Taken together, these findings indicate that EVA1A negatively regulates the plasma membrane localization of CD36.

### EVA1A deficiency promotes the palmitoylation of CD36 by modulating the expression of ZDHHC4/ZDHHC5 and APT1

**Fig. 10.**
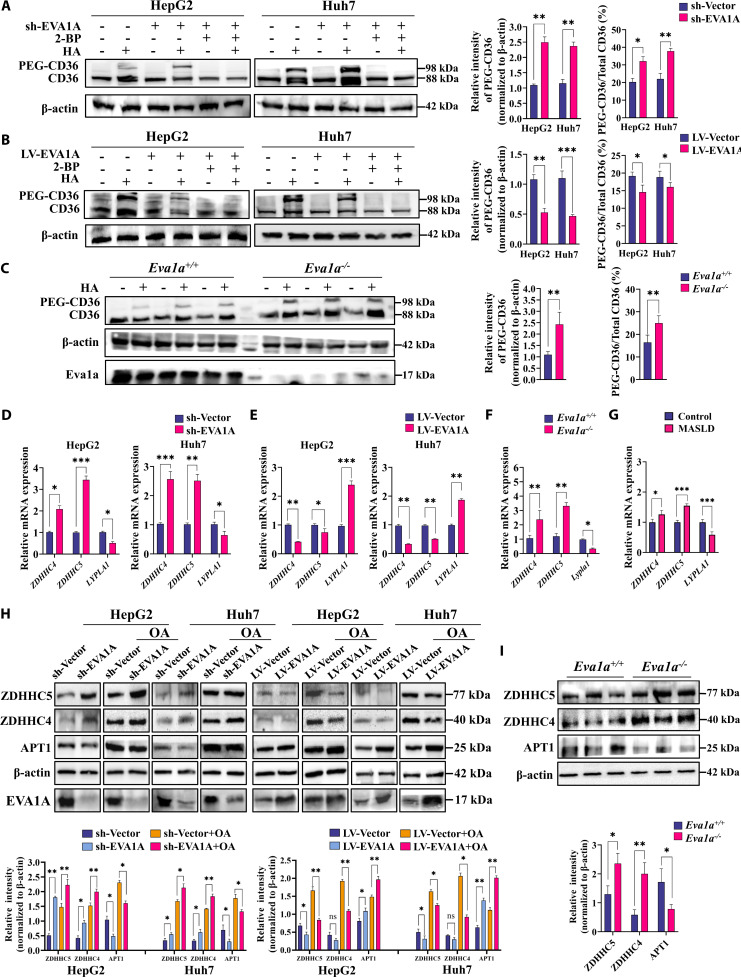
EVA1A deletion promotes the palmitoylation of CD36. (A to C) The palmitoylation levels of CD36 were assessed using the APE assay after treatment with 2-BP (50 μM, 6 h) as indicated, with or without HA. The top band indicated the palmitoylated CD36 (PEG-CD36). Detection of palmitoylated CD36 in EVA1A-knockdown HepG2 and Huh7 cells, along with control cells (A). Detection of palmitoylated CD36 in EVA1A-overexpressed HepG2 and Huh7 cells or control cells (B). Detection of palmitoylated CD36 in live tissues of *Eva1a^+/+^* mice and *Eva1a^−/−^* mice (*n* = 3) (C). Protein levels of palmitoylated CD36 were quantified in the right panels. (D to G) Relative mRNA levels of genes related to palmitoyl transferases (Z*DHHC4* and Z*DHHC5*) and acyl-protein thioesterase 1 (*LYPLA1*) in vitro (D and E) and in vivo (F and G). (H) Western blot analysis of ZDHHC5, ZDHHC4, and APT1 in EVA1A-knockdown or overexpressed HepG2 and Huh7 cells, after 6-h treatment with 400 μM OA or after being left untreated. (I) Western blot analysis of ZDHHC5, ZDHHC4, and APT1 in liver tissues of *Eva1a^+/+^* mice and *Eva1a^−/−^* mice (*n* = 3). Their protein levels were quantified in the lower panels. *P < 0.05, **P < 0.01, ***P < 0.001. HA: NH_2_OH.

Given that palmitoylation is critical for CD36 plasma membrane localization, we investigated whether EVA1A could influence the palmitoylation of CD36. To assess the palmitoylation levels of CD36, we conducted an acyl-PEG exchange (APE) assay. Palmitoylated CD36 was exchanged to PEG-CD36. Palmitoylation of endogenous CD36 was significantly up-regulated in EVA1A-knockdown HepG2 and Huh7 cells compared with control cells, as determined by Western blot analysis, whereas both EVA1A-overexpressing cell lines showed markedly reduced CD36 palmitoylation (Fig. 10A and B). Moreover, following treatment with the palmitoyl transferase pan-inhibitor 2-bromohexadecanoic acid (2-BP), which has been assessed for its capacity to induce cytotoxicity in HepG2 and Huh7 cells (Fig. S8), the palmitoylation levels of CD36 were dramatically diminished (Fig. 10A and B). Confirming our in vitro findings, the protein levels of palmitoylated CD36 were notably elevated in *Eva1a^−/−^* mice (Fig. 10C). Furthermore, omitting hydroxylamine (HA) abolished CD36 palmitoylation (Fig. 10A to C), confirming that CD36 is S-palmitoylated via thioester bonds. Since the palmitoyl group of CD36 is added by palmitoyl acyltransferases ZDHHC4 and ZDHHC5 [28], and deacylation is performed by acyl-protein thioesterases (APT1, gene name *LYPLA1*) [11], we further detected the expression of these enzymes. RT-qPCR and Western blot analyses revealed consistent up-regulation of ZDHHC4 and ZDHHC5 in EVA1A-knockdown cells; in contrast, APT1 expression was down-regulated at both the mRNA and protein levels (Fig. 10D and H). As expected, EVA1A overexpression produced nearly the opposite effects (Fig. 10E and H). Notably, although OA exposure markedly increased the expression of all these enzymes, the regulatory effects of EVA1A expression persisted (Fig. 10H). Furthermore, consistent results were obtained in vivo. EVA1A knockout markedly up-regulated the expression of ZDHHC4 and ZDHHC5, while significantly down-regulating APT1 at both the mRNA and protein levels (Fig. 10F and I). More importantly, in the livers of MASLD patients, the transcriptional levels of these 3 enzymes also showed a similar expression pattern to that of the *Eva1a*-LKO mice (Fig. 10G). Together, these results indicate that EVA1A negatively regulates CD36 palmitoylation by promoting its depalmitoylation, through modulation of ZDHHC4/ZDHHC5 and APT1 expression.

### Inhibition of CD36 palmitoylation diminishes EVA1A deficiency-induced fatty acid uptake and lipid accumulation

**Fig. 11.**
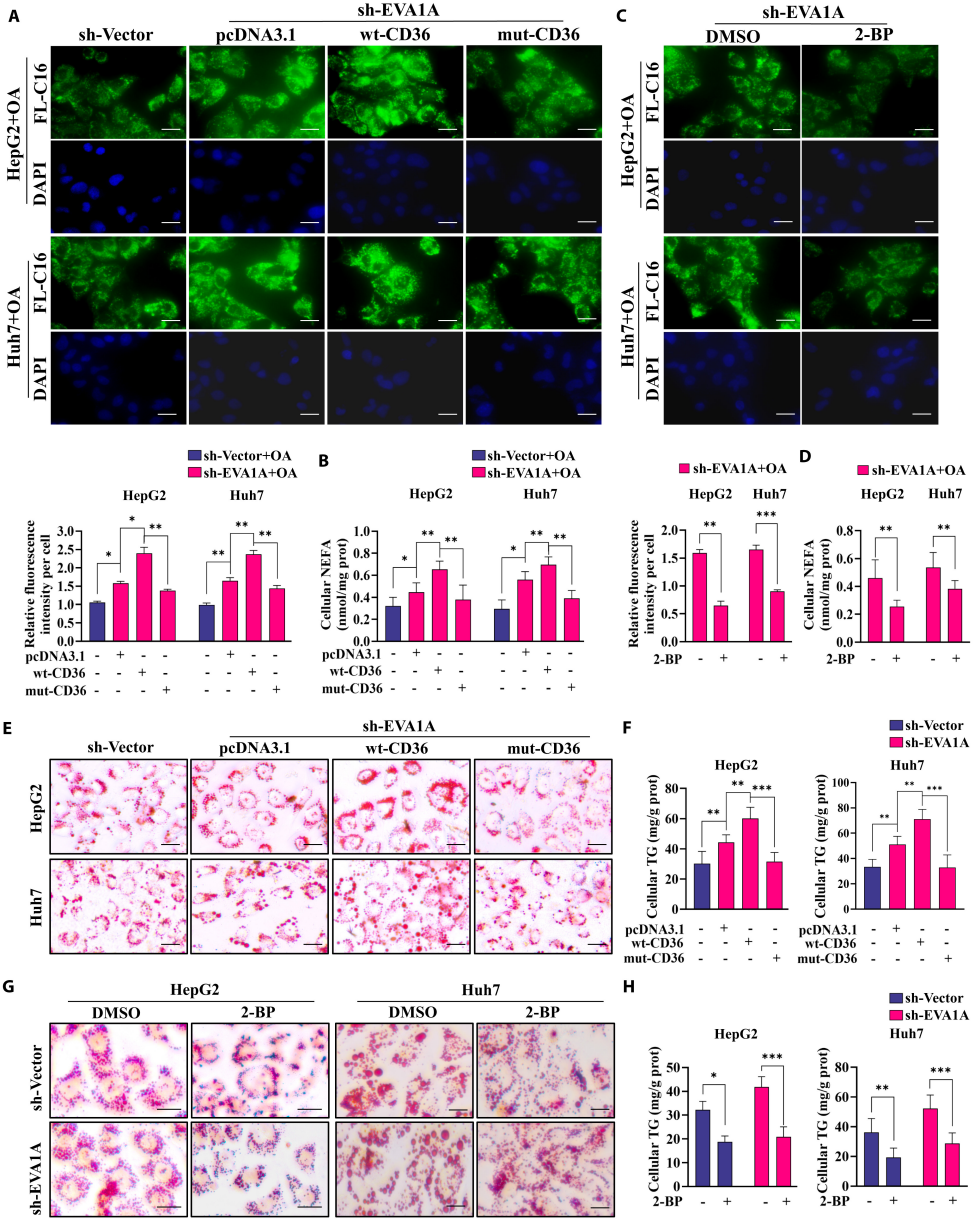
Inhibition of CD36 palmitoylation attenuates EVA1A deficiency-induced fatty acid uptake and lipid droplet accumulation. (A and E) The EVA1A-knockdown HepG2 or Huh7 cells were transfected with empty vector (pcDNA3.1) or wt-CD36, or mut-CD36 plasmid for 24 h, which had been treated with 400 μM OA during the final 6 h, then were fixed and stained with BODIPY FL-C16 (A) or ORO (E). Scale bars: 20 μm. The fluorescence signal intensity of FL-C16 per cell was quantified by ImageJ. Values were means ± SDs (*n* = 50). (B) Cellular NEFA levels in cells subjected to the treatment shown in (A). (C and G) The EVA1A-knockdown cells were co-treated with 50 μM 2-BP and 400 μM OA for 6 h, then were subjected to BODIPY FL-C16 staining (C) or ORO staining (G). Scale bars: 20 μm. (D) Cellular NEFA levels in cells subjected to the treatment shown in (C). (F) Cellular TG contents in cells treated as described in (A). (H) Cellular TG contents in cells treated as described in (C). *P < 0.05, **P < 0.01, ***P < 0.001.

Considering the critical role of the dynamic palmitoylation–depalmitoylation cycle in governing CD36 plasma membrane localization and modulating fatty acid uptake, we postulated that the palmitoylation of CD36 plays a contributory role in the enhanced fatty acid uptake and subsequent lipid droplet accumulation observed upon EVA1A depletion. To inhibit the palmitoylation of CD36 in EVA1A-knockdown HepG2 and Huh7 cells, we used 2-BP or overexpressed a mutant CD36 (mut-CD36) with a plasmid in which all 4 palmitoylation sites were mutated, thereby preventing any palmitoylation. Western blot analysis confirmed the overexpression of Flag-tagged wild-type CD36 (wt-CD36) or mut-CD36 (Fig. S9A). BODIPY FL-C16 staining revealed that, regardless of OA exposure, wt-CD36 significantly increased fatty acid uptake compared to pcDNA3.1-transfected controls, whereas mut-CD36 notably reduced fatty acid uptake in comparison to wt-CD36 (Fig. 11A and B and Fig. S9B and C). Furthermore, 2-BP treatment abolished the EVA1A deletion-induced increase in fatty acid uptake (Fig. 11C and D and Fig. S9D and E). Consistent with these findings, ORO staining and cellular TG measurement showed that wt-CD36 exacerbated both lipid droplet accumulation and cellular TG elevation following EVA1A deletion, whereas mut-CD36 (vs. wt-CD36) and 2-BP treatment significantly attenuated these effects caused by EVA1A deficiency (Fig. 11E to H). Based on these findings, we demonstrate that CD36 palmitoylation underlies the enhanced fatty acid uptake and lipid droplet accumulation upon EVA1A deletion.

### EVA1A enhances β-oxidation by promoting CD36 translocation to mitochondria

**Fig. 12.**
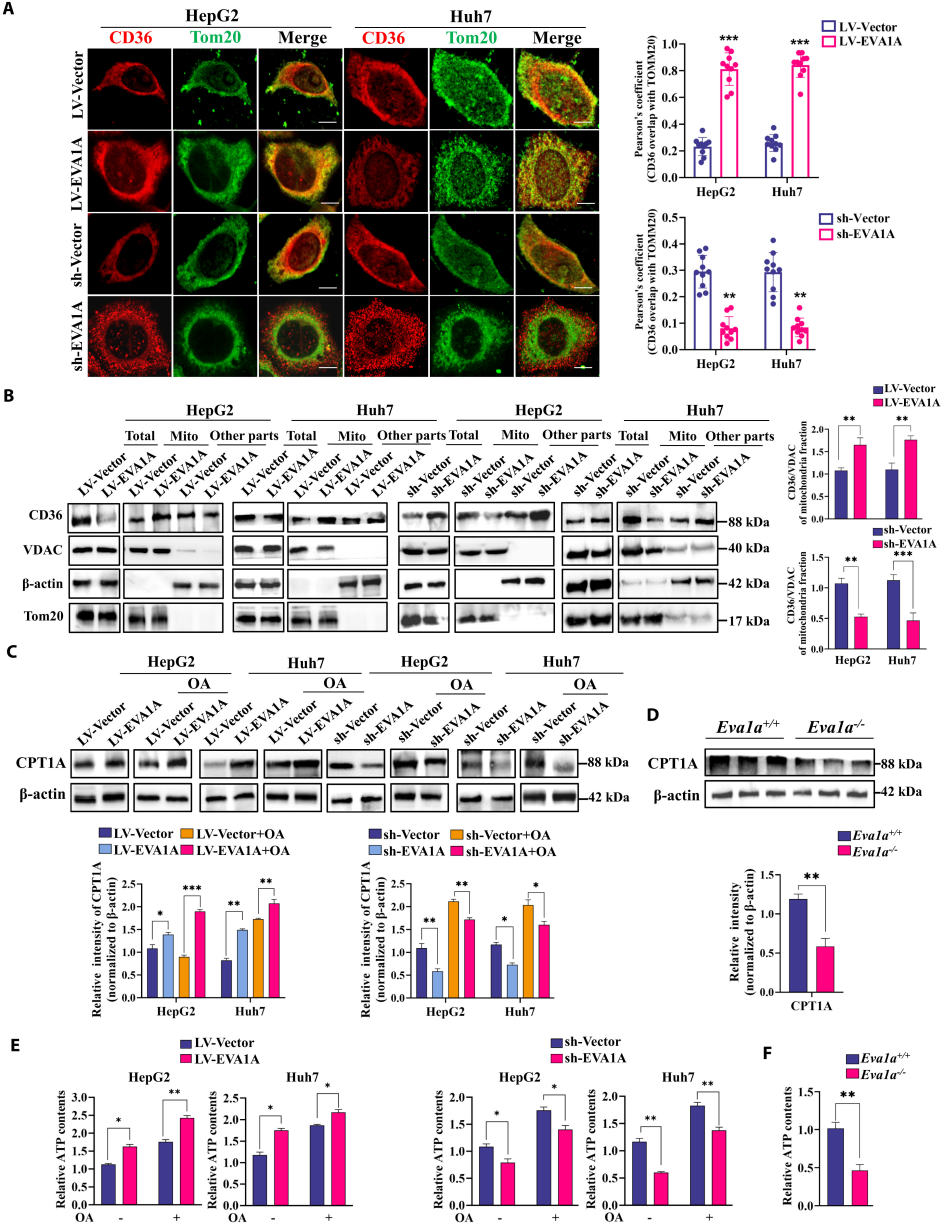
EVA1A facilitates CD36 mitochondrial localization and enhances β-oxidation. (A) EVA1A-overexpressing and EVA1A-knockdown HepG2 and Huh7 cells, along with their control cells, were immunostained with CD36 (red) and Tom20 (green) antibodies for confocal microscopy analysis. Scale bars: 5 μm. Colocalization analysis is shown in the right panels (*n* = 10). (B) Isolation the mitochondrial fractions and isolation the fractions other than nucleus and mitochondria. Analysis of CD36, VDAC, Tom20, and β-actin expression by Western blot. Mitochondrial CD36 levels were quantified in the right panels. (C) Western blot analysis of CPT1A in EVA1A overexpression or knockdown of HepG2 and Huh7 cells, in the presence or absence of OA (400 μM, 6 h) treatment. Their protein levels were quantified in the lower panels. (D) Western blot analysis of CPT1A in liver tissues of *Eva1a^+/+^* mice and *Eva1a^−/−^* mice. Protein levels were quantified in the lower panels (*n* = 3). (E) Relative ATP production in cells. (F) Relative ATP production in liver tissues of *Eva1a^+/+^* mice and *Eva1a^−/−^* mice. *P < 0.05, **P < 0.01, ***P < 0.001.

As the depalmitoylation of CD36 enables its translocation to mitochondria, thus promoting fatty acid β-oxidation [13], and EVA1A enhances CD36 depalmitoylation, we propose that EVA1A may boost fatty acid β-oxidation via facilitating CD36 mitochondrial localization, thereby playing a protective role against MASLD. We first monitored the colocalization of CD36 and mitochondrial Tom20 protein via confocal microscopy. As shown in Fig. 12A, CD36 exhibited strong colocalization with Tom20 in EVA1A-overexpressing HepG2 and Huh7 cells, but their colocalization was nearly absent in the EVA1A-knockdown cells. To further validate CD36 mitochondrial localization, we performed mitochondrial fractionation followed by immunoblotting for CD36. As shown in Fig. 12B, EVA1A overexpression substantially enhanced mitochondrial CD36 accumulation compared to control cells, while EVA1A knockdown significantly reduced CD36 levels in mitochondrial fractions. Given CD36’s role in transporting free fatty acids (FFAs) to mitochondria for β-oxidation, we sought to examine the expression of enzymes implicated in fatty acid β-oxidation and the associated transcription factors. RT-qPCR analysis showed that EVA1A overexpression significantly up-regulated *CPT1A*, *CPT2*, *ACADM*, *ACADL*, *HADHA*, *PPARGC1A* (encoding peroxisome proliferator-activated receptor gamma coactivator α [PGC1α]) and *PPARA* (encoding PPARα) mRNAs in HepG2 and Huh7 cells (Fig. S10). Western blot analysis demonstrated that EVA1A overexpression substantially increased CPT1A protein levels in both HepG2 and Huh7 cells, whereas EVA1A knockdown resulted in a significant decrease in CPT1A expression, independent of OA treatment (Fig. 12C). Consistent with the in vitro findings, the CPT1A protein expression in *Eva1a^−/−^* mice also decreased significantly compared to *Eva1a^+/+^* mice (Fig. 12D). Furthermore, we measured cellular adenosine triphosphate (ATP) levels in HepG2 or Huh7 cells under OA stimulation or not. The results indicated that EVA1A overexpression promoted ATP production, while EVA1A knockdown inhibited ATP production (Fig. 12E). Consistent with these findings, ATP production was markedly reduced in *Eva1a^−/−^* mice compared to *Eva1a^+/+^* mice (Fig. 12F). Taken together, these results indicate that EVA1A promotes the mitochondrial localization of CD36 and β-oxidation.

### Eva1a overexpression alleviates fatty liver in ob/ob mice

**Fig. 13.**
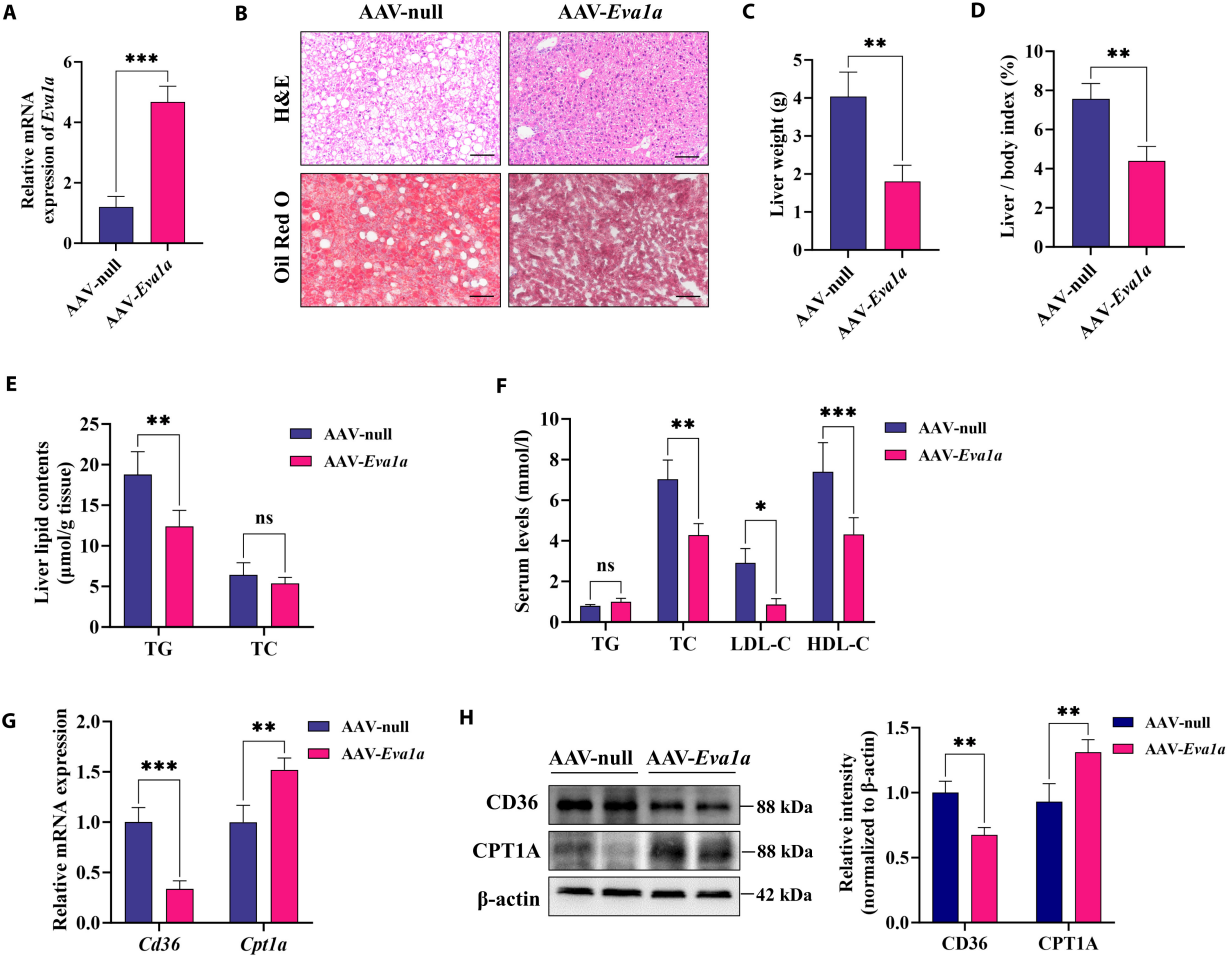
Eva1a overexpression alleviates fatty liver in ob/ob mice. (A) Hepatic *Eva1a* mRNA levels measured by RT-qPCR in ob/ob mice infected with AAV-*Eva1a* or AAV-null (*n* = 6). (B) Representative images of liver tissue from ob/ob mice stained with H&E and Oil Red O (*n* = 6). Scale bars: 100 μm. (C) The body weight of ob/ob mice (*n* = 6). (D) The liver index (liver-to-body weight ratios) of ob/ob mice (*n* = 6). (E) The liver TG and TC levels of ob/ob mice (*n* = 6). (F) Serum TG, TC, LDL-C, and HDL-C levels of ob/ob mice (*n* = 6). (G) The relative *CD36* and *Cpt1a* mRNA levels in ob/ob mice (*n* = 6). (H) Determination of CD36 and CPT1A protein expression by Western blot in ob/ob mice (*n* = 4). Protein levels were quantified in the right panels. *P < 0.05, **P < 0.01, ***P < 0.001.

To further validate the protective function of EVA1A against MASLD in vivo, we employed adeno-associated virus-mediated Eva1a overexpression in ob/ob mice, a well-established model of obesity and fatty liver. Hepatic Eva1a mRNA levels were significantly elevated in AAV*-Eva1a*-infected ob/ob mice compared with those receiving AAV-null (Fig. 13A). Eva1a overexpression markedly attenuated hepatocellular ballooning and suppressed hepatic lipid droplet accumulation (Fig. 13B). The AAV-*Eva1a* group showed significantly lower liver weight and a reduced liver-to-body weight ratio compared with controls (Fig. 13C and D), along with reduced hepatic TG content and decreased serum TC, LDL-C, and HDL-C levels (Fig. 13E and F). Contrary to the phenotype observed in *Eva1a-*LKO mice, Eva1a overexpression suppressed both transcription and expression of CD36, while enhancing those of CPT1A (Fig. 13G and H), thereby mitigating steatosis in the livers of ob/ob mice.

### The mTORC1–PPARγ2 signaling pathway mediates hepatic EVA1A deficiency-induced fatty acid uptake and lipid accumulation

**Fig. 14.**
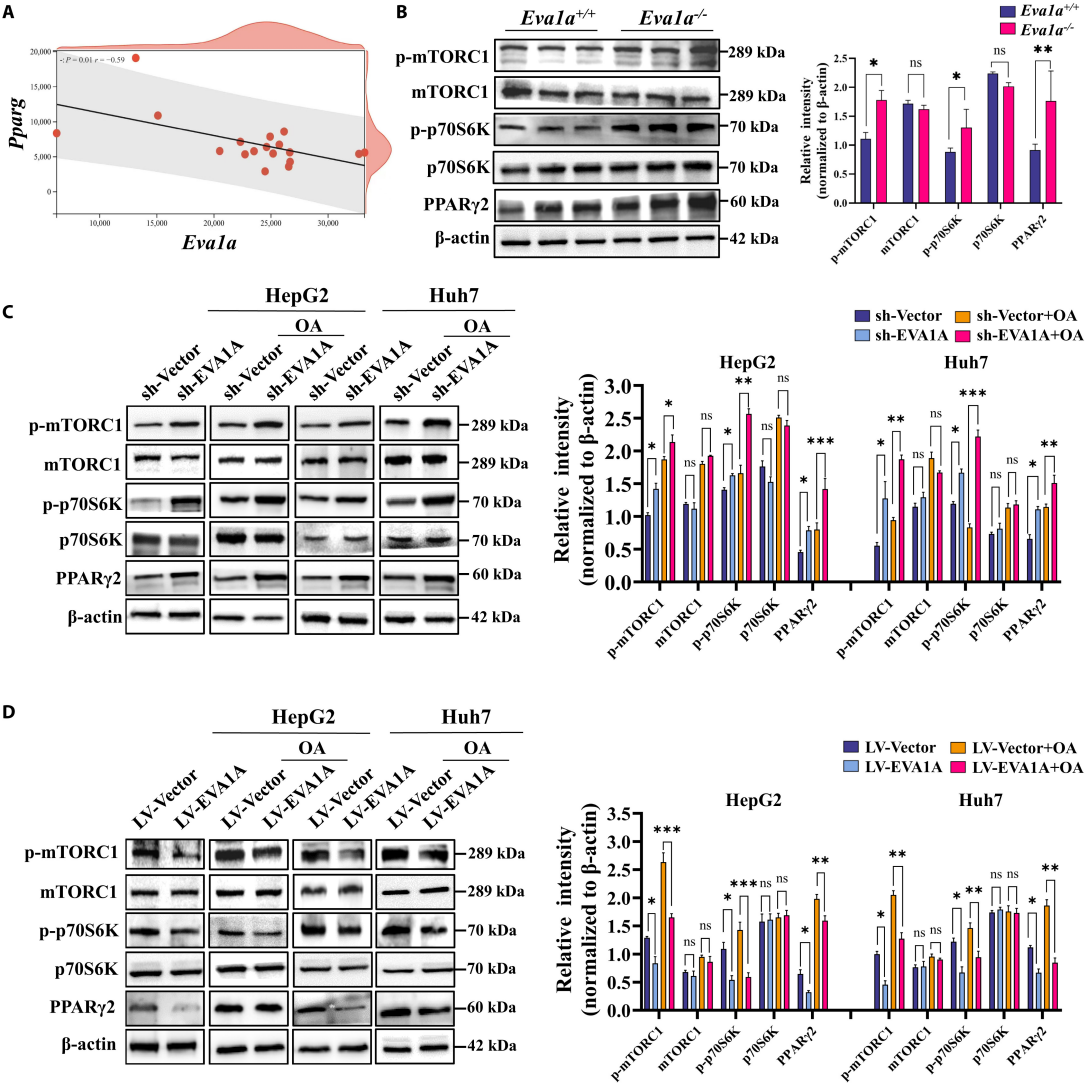
EVA1A negatively regulates the mTORC1–PPARγ2 signaling pathway. (A) *Eva1a* expression level was inversely correlated with *Pparg* expression level in the HFD-induced MASLD mouse liver according to the data from the GEO database (*P* = 0.01; Pearson correlation). (B) Western blot analysis for proteins of PPARγ2 and the mTOR/p70S6K signaling pathway in *Eva1a^+/+^* mice and *Eva1a^−/−^* mice (*n* = 3). (C and D) Western blot analysis of PPARγ2 and mTOR/p70S6K signaling pathway proteins in EVA1A-knockdown (C) or EVA1A-overexpressed (D) HepG2 and Huh7 cells treated with or without OA (400 μM, 6 h). Protein levels were quantified in the right panels. *P < 0.05, **P < 0.01, ***P < 0.001.

Next, we sought to elucidate the signaling pathways involved in EVA1A deficiency-induced enhancement of fatty acid uptake. According to the report, hepatic lipogenesis is mediated by mTORC1–PPARγ signaling, which involves the early-induced lipogenic transcription factor PPARγ2 [29]. Through direct or indirect means, PPARγ2 activates the expression of downstream genes associated with lipogenesis (e.g., *SREBP-1c* and *ACC*) and fatty acid uptake (e.g., *CD36* and *FABP4*) to regulate hepatic lipid homeostasis [30,31]. Bioinformatic analysis of hepatic gene expression profiles from HFD-induced MASLD mouse models in the GEO database uncovered a prominent inverse relationship between *Eva1a* and *Pparg* transcript levels (*P* = 0.01, *r* = −0.59; Fig. 14A). In *Eva1a*-LKO mice, we observed increased phosphorylation of mTORC1 and its downstream effector p70S6K, along with elevated PPARγ2 protein expression (Fig. 14B). Consistently, in HepG2 and Huh7 cells, whether subjected to OA or not, EVA1A knockdown significantly activated the mTORC1–PPARγ2 pathway; conversely, EVA1A overexpression markedly inhibited the mTORC1–PPARγ2 pathway (Fig. 14C and D). Collectively, our results identify EVA1A acts as a negative regulator of the mTORC1–PPARγ2 signaling pathway.

**Fig. 15.**
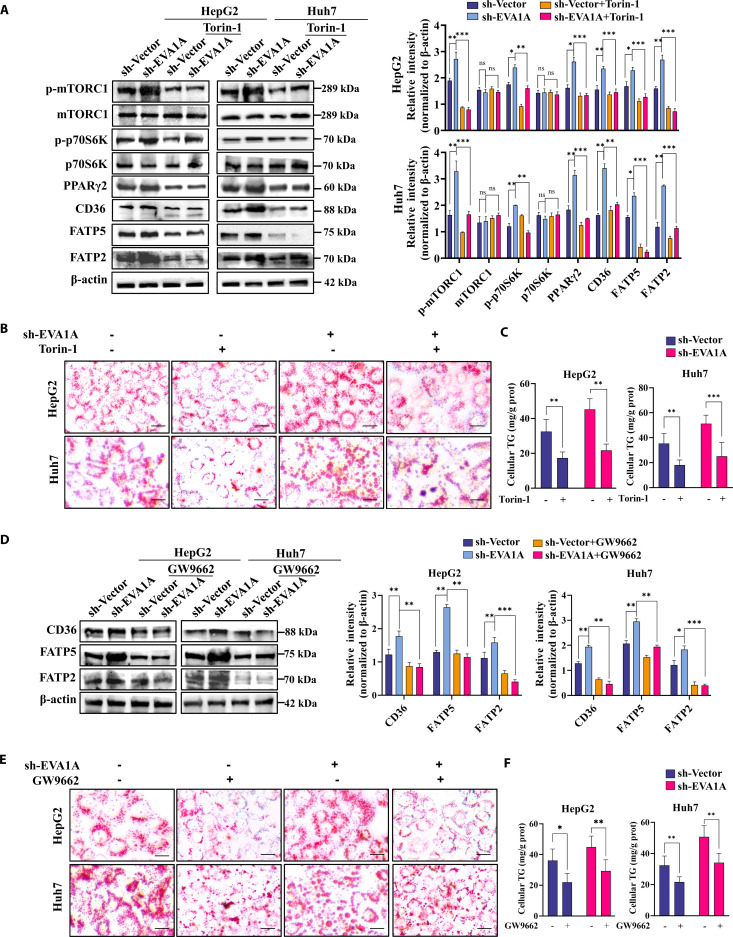
The mTORC1–PPARγ2 signaling pathway mediates EVA1A deletion-induced fatty acid uptake and lipid accumulation. (A and D) Western blot analysis for PPARγ2 protein, the mTOR/P70S6K signaling pathway (p-mTORC1 and p-P70S6K), and fatty acid transport proteins CD36, FAT5, and FATP2 in EVA1A-knockdown HepG2 and Huh7 cells and control cells with or without Torin1 (300 nM, 6 h) (A) or GW9662 (5 μM, 12 h) treatment (D). Protein levels were quantified in the right panels. (B and E) Representative microscopy images of ORO staining for lipid droplets in control or EVA1A-knockdown cells with or without Torin1 (B) or GW9662 (E) treatment. Scale bars: 20 μm. (C and F) The cellular TG contents upon Torin1 treatment (C) or GW9662 treatment (F) were quantified. *P < 0.05, **P < 0.01, ***P < 0.001.

To further investigate the vital role of the mTORC1–PPARγ2 signaling pathway in fatty acid uptake and lipid accumulation induced by EVA1A deficiency. We employed the mTORC1 inhibitor Torin-1 and the PPARγ antagonist GW9662 to block this pathway and assessed whether they could convert the phenotype of EVA1A-knockdown cells upon metabolic challenge. The cytotoxicity of these 2 reagents was first examined via a CCK-8 assay to determine the appropriate intervention duration and concentration (Fig. S11A and B). As shown in Fig. S11, Torin-1 had little effect on the viability of the HepG2 and Huh7 cell when its concentration was less than 300 nM and the duration was less than 6 h, while the corresponding parameters for GW9662 were 5 μM and 12 h. After treatment with Torin-1, the protein levels of p-mTORC1, p-p70S6K, and PPARγ2 were significantly reduced (Fig. 15A). Treatment with Torin-1 counteracted EVA1A deficiency-induced lipid droplet accumulation (Fig. 15B) and the increase in cellular TG levels (Fig. 15C). Concomitantly, it suppressed the expression of genes related to fatty acid uptake and transport (CD36, FATP5, and FATP2) at both the protein (Fig. 15A) and transcript (Fig. S12A) levels. Similarly, pharmacological inhibition of PPARγ2 by GW9662 reversed the impact of EVA1A deficiency on CD36, FATP5, and FATP2 expression (Fig. 15D and Fig. S12B), lipid droplet accumulation (Fig. 15E), and cellular TG augmentation (Fig. 15F). Collectively, our findings demonstrate that the mTORC1–PPARγ2 signaling pathway plays a pivotal role in regulating hepatic fatty acid uptake and lipid accumulation arising from EVA1A deficiency.

## Discussion

In this study, we discovered that EVA1A is a novel and essential regulator that maintains liver lipid balance through the mTORC1–PPARγ2–CD36 signaling axis. We demonstrate that EVA1A deficiency promotes hepatic lipid accumulation by simultaneously enhancing fatty acid uptake and suppressing β-oxidation, ultimately driving MASLD progression in mice. In contrast, overexpression of EVA1A restores metabolic balance by inhibiting fatty acid uptake and enhancing β-oxidation, thereby inhibiting MASLD progression in ob/ob mice. These findings define EVA1A as a key regulator of hepatic lipid metabolism and thereby identify it as a potential therapeutic target for MASLD.

**Fig. 16.**
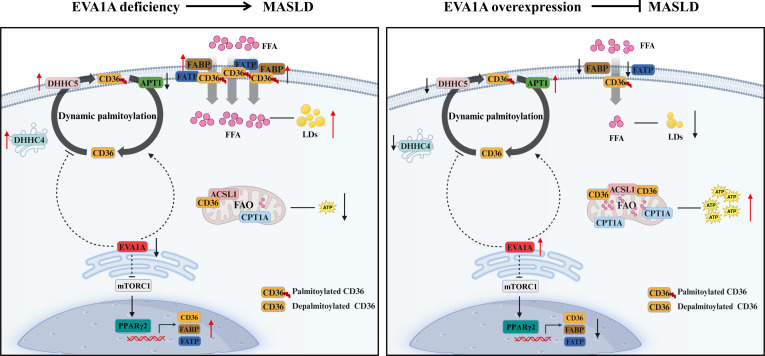
Schematic model of EVA1A regulating lipid metabolism in MASLD progression. Hepatic EVA1A expression levels influence MASLD progression by modulating the mTORC1–PPARγ2 signaling pathway and CD36 trafficking between the plasma membrane and mitochondria. EVA1A acts as an upstream inhibitor of mTORC1; its deletion activates the mTORC1–PPARγ2 signaling axis, up-regulating CD36 and other fatty acid transporters. In parallel, EVA1A deficiency increases the expression of palmitoyl transferases ZDHHC5 and ZDHHC4 while down-regulating the depalmitoylase APT1, which enhances CD36 palmitoylation and plasma membrane localization. This modulation enhances FFA intake, promotes lipid droplet accumulation in hepatocytes, and inhibits fatty acid β-oxidation, thus accelerating the progression of MASLD. In contrast, EVA1A overexpression inhibits mTORC1–PPARγ2 signaling axis and down-regulates CD36 and other fatty acid transporters. Meanwhile, EVA1A overexpression reduces the expression of ZDHHC5 and ZDHHC4 while up-regulating APT1 expression, promoting CD36 depalmitoylation and mitochondrial translocation. Consequently, FFA uptake and lipid droplet accumulation decrease, whereas fatty acid β-oxidation increases, which may attenuate MASLD progression. FFA, free fatty acid.

We constructed liver-specific *Eva1a* knockout mice and the mice develop hepatic steatosis. Gene expression linked to fatty acid uptake and lipid synthesis was markedly up-regulated, while gene expression associated with fatty acid catabolism was notably down-regulated. Genes linked to very low-density lipoprotein assembly and secretion, however, showed little change in expression. When exposed to OA to mimic high lipid loading, primary rat hepatocytes and 2 hepatoma cell lines, HepG2 and Huh7, showed a marked increase in fatty acid uptake and lipid accumulation following EVA1A deletion, along with a notable decrease in fatty acid β-oxidation and ATP generation. In contrast, EVA1A overexpression produced the opposite effects. Mechanistically, EVA1A was found to negatively regulate the genes’ expression connected to fatty acid uptake, including CD36 and other FATPs. Accordingly, the lipid accumulation and elevated fatty acid uptake induced by EVA1A deletion were completely abolished upon CD36 knockdown. Intriguingly, EVA1A deficiency not only elevated the expression of CD36 but also enhanced its palmitoylation, increasing its cell membrane localization but decreasing its mitochondrial localization. Conversely, EVA1A overexpression reduced CD36 palmitoylation and membrane localization but promoted its mitochondrial targeting, likely facilitating fatty acid β-oxidation and ATP generation. Using the palmitoylation inhibitor 2-BP or a CD36 palmitoylation mutant, we confirmed that CD36 palmitoylation mediates the cellular uptake of fatty acids and lipid accumulation induced by EVA1A deletion. Mechanistically, we found that EVA1A suppressed the mTORC1–PPARγ2 axis to inhibit CD36 transcription. Its deficiency up-regulated CD36 and palmitoyl transferases ZDHHC4/5 but down-regulated the depalmitoylase APT1; conversely, its overexpression produced opposing effects. The detailed mechanisms are shown in Fig. 16.

Numerous studies have recently demonstrated that CD36 is an essential regulator of fatty acid uptake, acting as an important contributor to MASLD development. Elevated hepatic CD36 levels are observed in HFD-induced MASLD models [12,32], while hepatocyte-specific CD36 deletion attenuates fatty acid uptake and protects against steatosis [33,34]. Consistent with these reports, our study demonstrated that CD36 ablation reverses the enhanced fatty acid uptake and lipid accumulation brought on due to EVA1A deficiency, highlighting CD36-dependent fatty acid uptake as the central mechanism in EVA1A deficiency-induced MASLD pathogenesis.

As a key fatty acid uptake transporter, CD36 is modulated by multiple posttranslational modifications, including glycosylation, ubiquitination, and palmitoylation [10]. In rat hearts, O-GlcNAcylation enhances fatty acid transport and oxidation by promoting CD36 plasma membrane localization [35]. In hepatocytes, however, it stabilizes CD36 by inhibiting ubiquitination, increasing its plasma membrane localization, and accelerating up the intake of fatty acids and lipid accumulation, thereby driving MASLD progression [14]. Polyubiquitination targets CD36 for proteasomal degradation, reducing fatty acid uptake [36]. The trafficking of CD36 to the plasma membrane and intake of lipids are both dependent on palmitoylation [11,13,28,37]. In macrophages, the palmitoylation of CD36 is regulated by oxidized high-density lipoprotein, thereby influencing lipid uptake [38]. Selenoprotein K enhances CD36 palmitoylation, facilitating its ER-to-Golgi transport via COPII vesicles. Palmitoylation inhibition disrupts CD36-COPII colocalization and improves hepatic steatosis [39]. Our study demonstrates that EVA1A deficiency, both in vivo (*Eva1a*-LKO mice) and in vitro (knockdown cells), increased the CD36 palmitoylation and its subsequent localization to the plasma membrane. In contrast, EVA1A overexpression reduced CD36 palmitoylation and promoted its translocation to mitochondria. These results establish EVA1A as a negative regulator of CD36 palmitoylation that determines its subcellular distribution. Notably, enhanced palmitoylation prolongs CD36 half-life [40], consistent with our finding that EVA1A deletion increases both CD36 palmitoylation and protein levels. Significantly, the palmitoylation inhibitor 2-BP reduced both CD36 palmitoylation and total protein levels (Fig. 10A and B), further demonstrating palmitoylation’s critical role in stabilizing CD36. Given the established role of glycosylation in promoting CD36 plasma membrane localization and stabilizing it by inhibiting ubiquitination [14], this study cannot exclude the possibility that EVA1A down-regulation also enhances CD36 GlcNAcylation. Moreover, whether EVA1A overexpression can—either directly or indirectly, by reducing CD36 GlcNAcylation—promote CD36 ubiquitination and subsequent degradation, thereby lowering its protein level, remains a key question for future investigation.

Palmitoyl transferases and S-depalmitoylases control the dynamic posttranslational alteration known as S-palmitoylation [36]. In adipocytes, ZDHHC4 catalyzes the palmitoylation of CD36 in the Golgi, creating a sorting signal, and ZDHHC5 retains CD36 at the plasma membrane by inhibiting its depalmitoylation. CD36 binds fatty acids and is depalmitoylated by APT1 before facilitating caveolar endocytosis to transport fatty acids into the cells [11,37]. EVA1A deficiency exhibited up-regulated hepatic ZDHHC4/5 and down-regulated APT1, while EVA1A overexpression showed opposite effects, indicating that EVA1A controls CD36 palmitoylation by transcriptionally regulating its modifying enzymes. However, due to the several number of these enzymes and the complexity of their regulatory networks preclude, the specific regulatory mechanisms of EVA1A for these enzymes and the potential impact on their activity have not been elaborated in this study.

It is reported that phosphorylation and acetylation regulate the activity of palmitoyl transferases or S-depalmitoylases [41]. In adipocytes, fatty acid-bound CD36 activates Src kinase LYN. LYN then phosphorylates ZDHHC5 at Tyr91 to inactivate it and promote CD36 depalmitoylation [11]. In diabetic cardiomyopathy, activation of Takeda G-protein-coupled receptor 5 (TGR5), a bile acid receptor, triggers cyclic AMP-protein kinase A (cAMP-PKA)-mediated phosphorylation and consequent inactivation of ZDHHC4. This sequence ultimately suppresses CD36 palmitoylation and its fatty acid uptake [42]. In melanoma, Wnt5a enhances APT1 activity through phosphorylation [43]. However, under high glucose conditions, the acetylation level of APT1 decreases, correlating with a reduction in its enzymatic activity [44]. Furthermore, both ZDHHC5 and APT1 can be modified by palmitoylation, which indirectly affects their substrate binding through regulating membrane localization [45,46]. For instance, DHHC5 palmitoylation and its acyltransferase activity can be inhibited by the reduction of the CD36/Fyn/Lyn complex caused by protocatechuic acid [45]. Whether EVA1A modulates these enzymes’ activity via similar mechanisms requires further investigation.

In the context of nutritional regulation, ZDHHC5 expression is modulated by fatty acid sensing, with its up-regulation in the livers of mice with metabolic dysfunction-associated steatohepatitis being driven by a fatty acid-activated JNK/c-Jun axis that directly enhances ZDHHC5 transcription [47]. Another example is that docosahexaenoic acid reduces the production of endogenous fatty acids by down-regulating fatty acid synthase (FASN) expression, thereby inhibiting ZDHHC5 [48]. Besides, ZDHHC5 can be transcriptionally up-regulated by mutant p53 along with the nuclear transcription factor NF-ϒ in glioma [49], but liver X receptor (LXR) stimulation decreases its mRNA and protein levels in Michigan Cancer Foundation-7 cells [35]. APT1 primarily senses glucose signals. In Type 2 diabetic β-cells, despite an increase in APT1 mRNA levels, its enzymatic activity is reduced [50]. This inverse relationship suggests that hyperglycemia may suppress APT1 activity through a posttranslational mechanism, such as deacetylation [44]. Moreover, miR-138 could inhibit APT1 translation in human umbilical vein endothelial cells [51]. Based on the above, we speculate that EVA1A may suppress ZDHHC5 transcription via JNK/c-Jun inhibition or LXR activation and counteract the inhibitory effect of miR-138 on APT1, which warrants definitive validation in future studies.

The regulatory effect of EVA1A on the depalmitoylation of CD36 prompted us to further investigate its function in fatty acid catabolism. Mitochondria are the main sites for hepatic fatty acid β-oxidation [52,53]. By employing fluorescence colocalization studies and mitochondrial protein extraction, we quantitatively analyzed CD36 expression. The findings showed that CD36 levels were notably reduced in the mitochondria of EVA1A-knockdown cells, whereas its mitochondrial expression was markedly increased in EVA1A-overexpressing cells. This observation aligns with a 2022 study, which suggested that inhibiting CD36 palmitoylation decreases long-chain fatty acid (LCFA) uptake, promotes CD36 localization to the mitochondria, and enhances its interaction with acyl-CoA synthetase long-chain family member 1. Through this interaction, LCFAs are converted to acyl-CoA to fuel the β-oxidation process within hepatocytes, thereby mitigating lipid accumulation in MASLD [13]. Therefore, the fatty acid utilization rate can be assessed by evaluating the efficiency of β-oxidation. Consistent across in vitro and in vivo models, we found that EVA1A deletion significantly reduced key enzyme CPT1A expression and ATP production. Conversely, EVA1A overexpression increased CPT1A expression and ATP generation. These findings underscore EVA1A’s regulatory function in hepatic fatty acid catabolism and its potential protective effect against MASLD.

To clarify the mechanism by which EVA1A triggers CD36 and other FATPs expression, we detected the mTORC1–PPARγ2 signaling pathway. There is compelling evidence that mTOR signaling regulates the balance between lipid synthesis and oxidation across tissues [54]. In adipocytes, mTORC1 controls PPARγ activation transcriptionally and mediates PPARγ-induced fatty acid uptake [55,56]. In hepatocytes, PPARγ activation, indicated by increases in PPARγ protein and mRNA, is associated with its interaction with raptor, a key mTORC1 component, mediating hepatic lipogenesis in ghrelin-infused mice [28]. Inhibition of the mTORC1–PPARγ pathway diminishes hyperlipidemia and hepatic steatosis in hamsters and mice [55]. In mesenchymal stem cells, this pathway regulates lipid metabolism and biosynthesis of lipid droplets [57]. PPARγ2, a nuclear hormone receptor, is up-regulated both in OA-treated hepatocytes and in the livers of HFD-fed mice. It regulates lipid metabolism by modulating downstream factors engaged in fatty acid uptake (CD36 and the FATP family) and de novo fatty acid synthesis (SREBP1 and SCD1) [58,59]. The development of MASLD is tightly linked to PPARγ2. A study published in 2023 indicated that GW9662, a PPARγ antagonist, alleviated hepatic steatosis by suppressing the PPARγ2–CD36 pathway, consequently slowing the progression of MASLD [60]. Herein, we found that EVA1A deletion activated the mTORC1–PPARγ2 signaling axis, verified by raised expression of p-mTOR and its downstream target, p-p70S6K, along with elevated levels of PPARγ2 transcripts and proteins. In contrast, EVA1A overexpression reduced p-mTOR and p-p70S6K levels, as well as PPARγ2 transcript and protein levels. These results confirm EVA1A’s negative regulatory role on the mTORC1–PPARγ2 pathway. Pharmacological inhibition of mTOR signaling with Torin-1 and PPARγ2 signaling with GW9662 effectively restored the altered expression of PPARγ2, CD36, and FATPs, and rescued the abnormal lipid accumulation phenotype caused by EVA1A deficiency. These findings verified that EVA1A regulates crucial factors engaged in fatty acid uptake, such as CD36, through the mTORC1–PPARγ2 pathway, thereby influencing fatty acid uptake and lipid droplet accumulation, which affects MASLD onset and progression.

Although the precise mechanisms by which EVA1A regulates fatty acid catabolism require further investigation, our findings unequivocally indicate the critical involvement of the mTOR signaling pathway in this process. Specifically, EVA1A overexpression suppressed mTORC1 activity (Fig. 14D) while simultaneously up-regulating PPARα and PGC-1α expression (Fig. S10). This is particularly significant given the well-established function of the PPARα/PGC-1α pathway in activating hepatic fatty acid oxidation genes, including CPT1A [61]. Under fasting conditions, mTORC1, via its interaction with its downstream S6K2, promotes nCoR1 nuclear accumulation, thereby attenuating PPARα activity in the liver [62,63]. In cultured cardiomyocytes, the activation of mTORC1 attenuated PGC1α–PPARα-mediated transcription of β-oxidation and mitochondrial electron chain factors [64]. Consequently, it is highly probable that the EVA1A promotes β-oxidation through the mTORC1–PPARα/PGC1 signaling pathway. Notably, our study cannot exclude the potential contributions of lipogenesis to EVA1A deficiency-mediated MASLD progression, as evidenced by up-regulated transcription of lipogenic enzymes and the key transcription factor *Srebf1* in *Eva1a*-LKO mice (Fig. 3), although this effect may represent a secondary consequence of enhanced fatty acid uptake. Lipogenic genes in the liver are primarily regulated by the lipogenic transcription factor sterol regulatory element-binding protein 1c (SREBP1c) and both the transcriptional activity and expression level of SREBP1c are positively regulated by mTORC1. Studies have shown that the mTORC1 signaling pathway promotes SREBP1c transcription, translation, and proteolytic processing, facilitating its maturation and thus regulating lipogenesis [54,65]. Whether down-regulation of EVA1A can promote lipid synthesis during the progression of MASLD by activating the mTORC1–SREBP1c pathway requires further investigation.

Regarding the mechanism by which EVA1A regulates mTORC1 activity, there are currently 2 plausible hypotheses. First, it may occur through the PI3K–AKT pathway. Our recent study demonstrated that EVA1A negatively regulates the PI3K–AKT signaling pathway, contributing to lenvatinib resistance in HCC [20]. Given that mTORC1 acts downstream of this pathway and is positively regulated by it, EVA1A-mediated suppression of PI3K–AKT could consequently inhibit mTORC1 activation. Second, mTORC1 is activated on the lysosomal membrane in response to amino acid signaling. Intracellular amino acid sufficiency promotes their accumulation within lysosomes, triggering the v-ATPase-Regulator complex to transmit signals to Rag GTPases. This process activates Rag GTPases, leading to the recruitment of mTORC1 to the lysosomal membrane, where it is subsequently activated by Rheb anchored there [66,67]. Notably, EVA1A is partially localized to lysosomes and may function by modulating the activity of these small GTPases or acting as an inhibitory factor that prevents mTORC1 recruitment to the lysosomal membrane.

Recently, numerous studies have shown that lipophagy is crucial for maintaining the lipid homeostasis of the liver and lipophagy is impaired in MASLD [68,69]. Given that EVA1A is a regulator of autophagy [15], we cannot exclude whether EVA1A regulates lipid metabolism through lipophagy. In the fatty liver tissues of *Eva1a*-LKO mice, we observed significant accumulation of p62 protein. Similarly, in EVA1A-knockdown cells, accumulation of LC3 (data not shown) and lipid droplets was also detected, suggesting that impaired autophagy may contribute to lipid droplet accumulation. Concurrently, the observed activation of mTORC1 in EVA1A-deficient mouse liver tissues and cells indicates suppression of upstream autophagic signaling. Conversely, EVA1A overexpression inhibited mTORC1 activity—an effect that would be expected to initiate autophagy—and was accompanied by markedly reduced lipid droplet accumulation. These preliminary findings imply that EVA1A may facilitate hepatic lipid clearance through mTORC1 inhibition-mediated lipophagy. In the field of lipophagy research, the specific recognition between lipid droplets and autophagosomes represents a current research focus [70–72]. Given that the endoplasmic reticulum serves as the site for both lipid droplet synthesis and autophagosome precursor formation, and considering EVA1A’s identity as an endoplasmic reticulum-associated protein, we hypothesize that EVA1A may participate in the recognition process between autophagosomes and lipid droplets, which constitutes a key interest for our future investigations.

Previous studies have identified several key regulators in hepatic lipid metabolism. For instance, TRIM56 acts as an endogenous negative regulator through direct interaction and promotion of FASN degradation [73]. CRTC2 reduces lipid synthesis by competitively inhibiting the Sec23A–Sec31A interaction, thus disrupting COPII-mediated transport of SREBP1 and its subsequent maturation [74,75]. Fat-1, an enzyme, is in charge of converting endogenous omega-6 polyunsaturated fatty acids (PUFAs) into omega-3 PUFAs and alleviates MASLD by enhancing PPARα-mediated fatty acid oxidation [76]. Moreover, microRNA-125b-5p is down-regulated in FFA-induced L02 cells, leading to de-repression of SREBP1, ACC1, and FAS and promoting lipid anabolism [77]. Distinct from these mechanisms, which primarily focus on lipid synthesis, our research employed a more comprehensive screening strategy encompassing exogenous fatty acid uptake, lipid synthesis, and β-oxidation. This enabled the identification of EVA1A as a pivotal regulator of overall hepatic lipid homeostasis and a critical target for treating fatty liver disease.

Based on the findings of this study, EVA1A down-regulation represents a key initiating event in abnormal lipid metabolism in MASLD patients and animal models. Unfortunately, the regulatory mechanisms controlling EVA1A expression in the context of fatty liver has not been revealed in this investigation. Preliminary data demonstrate that exposure to high FFAs mimicking the MASLD lipid-rich environment significantly reduces EVA1A mRNA levels, while returning cells to normal culture conditions restores its expression to baseline (Fig. S13A). In contrast, high glucose stimulation does not alter EVA1A RNA levels (Fig. S13B), suggesting that EVA1A expression specifically responds to circulating lipids rather than common sugars, despite the potential development of hyperglycemia and insulin resistance in advanced MASLD stages.

Furthermore, while the responsiveness of EVA1A to insulin signaling remains unclear, existing evidence indicates that EVA1A inhibits the PI3K/AKT pathway [20], suggesting a potential antagonistic role in insulin signal transduction. Down-regulation of EVA1A was also observed in leptin-deficient ob/ob mice (data not shown), indicating that its expression is independent of leptin signaling. In *Eva1a*-LKO mice, levels of inflammatory factors such as TNFα and IL-6 were comparable to controls (data not shown), further suggesting that EVA1A expression is not modulated by inflammatory cytokines. Moreover, both *Eva1a*-LKO mouse fatty liver tissues and EVA1A-knockdown cells exhibited impaired autophagic flux, indicating that reduced EVA1A protein levels are not attributable to autophagic degradation. Regarding the transcriptional regulation of EVA1A, we propose 2 mechanistic hypotheses. First, based on our recent findings that miR-103a down-regulates EVA1A and promotes hepatocellular carcinogenesis [18], and given that miR-103a is up-regulated in HFD-fed rats, we speculate that, in MASLD [78], elevated miR-103a may target and suppress EVA1A transcription. Second, lipid overload may activate histone deacetylases or dysregulate DNA methyltransferases, leading to transcriptional repression via histone deacetylation or promoter hypermethylation [79,80]. EVA1A expression may be subject to such epigenetic mechanisms.

## Conclusion

This research identifies EVA1A as a novel master regulator of hepatic lipid homeostasis through dual metabolic control. First, EVA1A governs fatty acid uptake by modulating both the expression and palmitoylation status of CD36. Second, EVA1A enhances fatty acid β-oxidation by up-regulating key enzymes (e.g., CPT1A) and promoting mitochondrial localization of CD36. Mechanistically, EVA1A controls the expression of CD36 and other FATPs through the mTORC1–PPARγ2 signaling axis. It modulates the dynamic palmitoylation of CD36 through balanced expression of palmitoyl acyltransferases ZDHHC4/5 and S-depalmitoylase APT1. This precise regulation determines CD36 subcellular distribution (plasma membrane vs. mitochondria), thereby balancing fatty acid uptake against oxidation. Our findings not only elucidate EVA1A’s central role in MASLD pathogenesis but also validate the EVA1A–CD36 regulatory axis as a viable therapeutic target against metabolic liver diseases.

## Materials and Methods

### Human samples

Human fatty liver samples were collected from biopsy-confirmed MASLD patients following the exclusion of other liver diseases. Control samples were from individuals without MASLD undergoing liver surgery. All participants had no history of drug abuse, hepatitis virus infection, or excessive alcohol consumption. The study received approval from the Medical Ethics Committee of the Affiliated Hospital of Qingdao University (approval no. QYFY WZLL 30204) and adhered to the principles of the Declaration of Helsinki.

### Animal experiments

Liver-specific *Eva1a* knockout mice (*Eva1a^−/−^*) were generated via the Cre/Loxp system, and the primers for identification are listed in the Supplementary Materials. We acquired *Alb-Cre* mice from Shanghai Southern Model Biology Co., Ltd. (Shanghai, China), and Professor Yingyu Chen (Peking University, China) donated *Eva1a^flox/flox^* mice. Male *Eva1a^+/+^* and *Eva1a^−/−^* mice, aged 6 weeks, were housed under specific-pathogen-free conditions with a 12-h light/dark cycle and provided with food and water ad libitum. We monitored weekly changes in body weight and food intake. Following a 16-week period, the mice were euthanized, blood samples were obtained, and the livers were dissected for immediate fixation in paraformaldehyde or stored in liquid nitrogen for future use.

Liver sections of MASLD mice were taken from an HFD-induced model, previously established by our group. The construction process is as follows: Male mice aged 6 weeks were randomly assigned to 2 groups and subjected to distinct dietary regimens: an NCD (10% fat, 72% carbohydrate, and 18% protein; XTM07-009; Xietong Bioengineering, China) or an HFD (60.39% fat, 20.56% carbohydrate, and 19.05% protein; XTM01-002; Xietong Bioengineering, China) for 16 weeks.

Male C57BL/6J ob/ob mice (8 weeks old, 40 to 45 g), a genetic model of obesity and fatty liver disease, were sourced from Jiangsu Jicui Yaokang Biotechnology Co., Ltd. and maintained in an SPF-level animal facility and fed standard chow. We randomly allocated the mice to receive an intravenous tail vein injection of either a serotype 8 AAV empty vector (AAV-*null* group) or recombinant vector AAV-*Eva1a* (AAV-*Eva1a* group). Following a 7-week intervention period, the mice were fasted overnight, anesthetized, and subjected to blood collection via the retro-orbital plexus. Thereafter, we euthanized the mice by cervical dislocation and then collected liver tissues for analysis.

All animal experiments were performed in accordance with ethical guidelines and were approved by the Ethics Committee of the Medical College of Qingdao University (approval no. QDU-AEC-2024786 and QDU-AEC-2024767).

### Isolation of primary rat hepatocytes

Male Sprague–Dawley rats (6 to 8 weeks old, 200 to 250 g) were used to isolate primary rat hepatocytes. The isolation procedure was performed utilizing a 2-step in situ collagenase perfusion technique adapted from Seglen [81]. Briefly, after anesthetizing the rats, the abdomen was opened, and the hepatic portal vein was punctured with an indwelling needle. The liver was initially perfused with 37 °C pre-warmed D-Hanks solution (BOSTER, China) containing 0.5 mM EDTA (Solarbio, China) and 25 mM HEPES (Solarbio, China) to clear blood, while the inferior vena cava was cut to facilitate drainage. When the liver turned wax-yellow, perfusion was switched to collagenase IV (Arcegen, China) until the liver became soft. The digested liver was excised, placed in 20 ml of ice-cold high-glucose Dulbecco’s Modified Eagle’s Medium (DMEM) (BasalMedia, China) containing 1% penicillin–streptomycin (P/S) (Solarbio, China), gently torn into pieces with forceps, and passed through a 100-μm cell strainer. The filtrate was centrifuged at 50×*g*, at 4 °C for 3 min; this wash was repeated 3 times to enrich hepatocytes. The cell pellets were resuspended and mixed with an equivalent amount of 4% Percoll (Solarbio, China), then hepatocytes were purified by centrifuging at 400×*g*, at 4 °C for 10 min. The trypan blue staining method (Solarbio, China) was used to assess cell viability. Primary hepatocytes were seeded at an appropriate density onto collagen-coated (Solarbio, China) 6-well plates and cultured in high-glucose DMEM medium containing 10% fetal bovine serum (MRC, Australia) and 1% P/S. Plates were placed in a 37 °C, 5% CO₂ incubator and the medium was BasalMedia changed 4 h after seeding.

### Cell lines, reagents, and plasmids

Pricella (Wuhan, China) provided the HepG2 and Huh7 cell lines. Cells were cultured in DMEM (BasalMedia, China) supplied with 10% fetal bovine serum (MRC, China) and 1% P/S (Solarbio, China) at 37 °C in a humidified 5% CO₂ incubator. For cell treatment, the final concentrations of OA (Sangon, China), 2-BP (MCE, USA), Torin-1 (Aladdin, China), and GW9662 (Aladdin, China) were 400 μM, 50 μM, 300 nM, and 5 μM, respectively. The plasmids encoding wt-CD36 (hCD36-C-FLAG/pCDNA3.1) and mut-CD36 with mutations in the palmitoylation sites of CD36 (C3S, C7S, C464S, and C466S) were generous gifts from Professor Tongjin Zhao (Xiamen University, China). The plasmids were delivered into sh-EVA1A cells with Lipofectamine 2000 (Invitrogen, USA).

### Stable cell line construction

Professor Yingyu Chen (Peking University, Beijing) generously provided 2 shEVA1A plasmids, which were employed for the knockdown assay. The targeted EVA1A sequences—shRNA1 (seq: GTGGATGGAGATCGGAGAAAC) and shRNA2 (seq: GAACCCAGCTCTGCTAATATT)—each encode a distinct short hairpin RNA (shRNA) sequences. The knockdown efficiency was validated for both individual shRNAs and the shRNA pool. We employed a lentiviral vector (Genechem, China) carrying EVA1A (LV-EVA1A) to generate stable EVA1A-overexpressing cell lines in both HepG2 and Huh7 cells. The pGC-FU-3FLAG-CBh-gcGFP-IRES-puromycin lentiviral vector (LV-vector) was used as a control. At 48 h posttransfection or infection, cells were treated with 3 to 5 μM puromycin (Beyotime, China) to select for stable knockdown or overexpressing cell lines.

### RNA interference

siRNAs against human CD36 (siCD36-1: 5′-GGCUGUGUUUGGAGGUAUUCUTT-3′, siCD36-2: 5′-CCCUGUUACUACCACAGUUTT-3′) and siRNAs against Rat Eva1a (siEva1a-1: 5′-ACGUUCUUGUUCAAAGUCCUCT-3′, siEva1a-2: 5′-GGACUUUGAACAAGAACGUGUT-3′) were obtained from GenePharma Corporation (Shanghai, China). Transfection procedures were carried out using Lipofectamine 2000 with the relevant siRNAs, as per instructions.

### Immunofluorescence and confocal microscopic analysis

After being fixed for 15 min with 4% paraformaldehyde (Biosharp, China), the cells were blocked for 1 h with goat serum (BOSTER, China). Subsequently, the cells were incubated at 4 °C with primary antibodies overnight: anti-CD36 (1:200, ABclonal/BOSTER, China), anti-Tom20 (1:200, Proteintech, China), or anti-ATP1A (1:800, Proteintech, China). The next step involved incubation with secondary antibodies conjugated to Alexa Fluor 488 (goat anti-rabbit) or Alexa Fluor 594 (goat anti-mouse immunoglobulin G [IgG]) (1:100, ABclonal, China) for 45 min at room temperature. The cells were finally incubated with DAPI (ABclonal, China) for 5 min for nuclear staining. The labeled cells were visualized and analyzed via a Ti2-U inverted fluorescence microscope (Nikon, Japan) or a confocal Stellaris 5 microscope (Leica, Germany). Representative images of CD36, Tom20, or ATP1A colocalization and the fluorescence intensity were analyzed via ImageJ.

### Immunohistochemistry

The liver samples underwent fixation in 4% paraformaldehyde for 1 day, subsequently embedded in paraffin for histological investigation, and sectioned at a thickness of 15 to 20 μm using a Leica CM1520 microtome (Germany). Firstly, antigen retrieval was performed by microwave heating of tissue sections in citrate buffer (pH 6.0). Then, the slices were incubated with either an anti-EVA1A antibody (1:200, ABclonal, China) or a CD36 antibody (1:100, BOSTER or 1:500, Proteintech, China) overnight at 4 °C. Normal IgG from rabbits or mice was used as the negative control. After a phosphate-buffered saline (PBS) wash, the sections were treated with a biotinylated secondary antibody followed by a streptavidin-horseradish peroxidase (HRP) assay. DAB (ZSGB-BIO, China) was used for cytochemical reactions, and hematoxylin was used as a counterstain for the samples. The neutral gum-sealed slices were allowed to dry before images were acquired via a Ti2-U inverted microscope. All images were acquired under the same conditions.

### Flow cytometric analysis of surface CD36

The cells were harvested by trypsinization (Solarbio, China), then centrifuged and washed with PBS. After incubation with the primary antibody APC-CD36 (1:100, Abcam, USA) for 1 h, fluorescence was detected via an Accuri C6 (BD Biosciences, USA) flow cytometer, and fluorescence intensity was analyzed via FLOWJo V9 software.

### RT-qPCR analysis

Total RNA was isolated from liver tissues or HepG2/Huh7 cells with TRIzol (Solarbio, China), following the manufacturer’s protocol. Complementary DNA was generated via a cDNA reverse transcription kit (Vazyme, China). Then, we used a SYBR Green Kit (Vazyme, China) to measure the relative mRNA expression levels of the target genes on a QuantStudio5 Real-Time quantitative PCR system (Thermo Fisher Scientific, USA), with normalization to β-actin expression. Tables S2 to S5 detail the primer sequences employed for gene identification.

### BODIPY 493/503 and BODIPY FL-C16 staining

A BODIPY493/503 Kit (Beyotime, China) was utilized for lipid droplet staining, while a BODIPY FL-C16 Kit (Thermo Fisher Scientific, USA) was employed for fatty acid staining. After fixation with 4% paraformaldehyde for 15 min, cells were washed thrice with PBS. Lipid droplets in cells were then stained by incubation with BODIPY 493/503 working solution for 30 min at room temperature in the dark. For the purpose of fatty acid staining, live cells were initially washed with PBS and subsequently incubated in a serum-free medium containing 2 μM BODIPY FL C16 for 30 min at 37 °C. After a brief PBS rinse, cells were fixed in 4% paraformaldehyde for 15 min and observed. Imaging was performed with an IX83 inverted microscope (Olympus, Japan), followed by quantification of fluorescence intensity using ImageJ software.

### Mitochondria and cell membrane fractionation

Cells (5 × 10^6^) cultivated in 25-cm trays were washed twice with ice-cold PBS and subsequently treated on ice with 2 ml of extraction buffer containing 5% mannose and sucrose and then were collected using a scraper. To remove the nuclei, the resuspended cells were centrifuged at 600×*g* for 5 min at 4 °C after being homogenized 30 to 50 times on ice using a homogenizer. Then, the collected supernatants were centrifuged at 10,000×*g* for 30 min at 4 °C. The precipitate was the mitochondrial fraction, and the other parts excluded the nucleus and mitochondria. For cell membrane extraction, under the premise of removing the cell nucleus and mitochondria, the supernatant of the removed mitochondria was centrifuged at 4 °C and 16,000×*g* for 30 min, after which the centrifugation and precipitation were repeated to obtain the cell membrane fragments. The cell membrane or mitochondrial proteins were extracted and then analyzed via Western blotting.

### Protein palmitoylation analysis by APE

The APE assay [82] was carried out following a previously published protocol with minor modifications. Briefly, after being washed thrice with PBS and trypsinized, cells were lysed in a buffer containing a protease inhibitor cocktail and 5 mM EDTA. For reduction and alkylation, the cell lysates were first incubated with 10 mM Tris(2-carboxyethyl)phosphine (Sangon, China) for 30 min. Prior to alkylation, the samples were then incubated with 25 mM N-ethylmaleimide (Aladdin, China) for 2 h at room temperature. Methanol, chloroform, and distilled water were added one after the other into a 1.5-ml Eppendorf tube in order to precipitate the combination. The protein pellets were rinsed 3 times with cold methanol and resuspended in triethanolamine (TEA) buffer containing 4% SDS. Prior to incubation, the resuspended pellets were mixed with 0.75 M NH₂OH (MCE, USA) and placed in a rotary mixer at room temperature overnight. After removing NH_2_OH with methanol–chloroform–water precipitation, the protein pellets were resuspended in TEA buffer containing 0.2% Triton X-100 and subsequently treated with 1 mM mPEG-Mal (10 kDa) (Ponsure, China) for 2 h at room temperature. Following a second round of methanol–chloroform–water precipitation, the samples were resuspended in nonreducing 1× Laemmli buffer, boiled for 3 min at 95 °C, resolved by sodium dodecyl sulfate–polyacrylamide gel electrophoresis (SDS-PAGE), and transferred for immunoblotting.

### Western blot analysis

Following lysis with radio immunoprecipitation assay (RIPA) buffer (Beyotime, China), proteins (40 to 100 μg per lane) from cells or mouse liver tissues were separated by 8%–12% SDS-PAGE and transferred to polyvinylidene fluoride (PVDF) membranes (Millipore, USA). After blocking with 5% nonfat milk in Tris-buffered saline with Tween-20‌ (TBST) for 3 h at room temperature, the membranes were incubated with specific primary antibodies at 4 °C overnight. After extensive washing with TBST, the membranes were subjected to incubation with HRP-conjugated secondary antibodies for 1 h at room temperature. After thorough washing, the membranes were incubated with enhanced chemiluminescence solution (Meilunbio, China) in the dark for 1 min, followed by image acquisition using a ProteinSimple imaging system (Bio-Techne, USA) and subsequent quantification with ImageJ software.

### Cell viability assay

Cell viability was detected utilizing the CCK-8 assay (Biosharp, China). Cells exposed to different concentrations of OA, 2-BP, Torin-1, or GW9662 were plated in 96-well plates. After varying incubation times, CCK-8 reagent (Biosharp, China) was added. After a 4-h incubation at 37 °C, the absorbance of the plates at 450 nm was noted with a microplate reader (Bio-Rad, USA). Cell viability was assessed utilizing GraphPad Prism 6 (Dotmatics, USA). For each experimental procedure, 3 independent replicates were performed.

### Liver and cellular TG and ATP-level assays

Liver lipid was extracted by the Folch method. In brief, 50 mg of mouse liver tissue was weighed or 1 × 10^6^ cells were collected and placed in an Eppendorf tube (containing steel beads). Add 1 ml of a chloroform/methanol (v/v, 2:1) mixture and rotate on a rotary mixer at room temperature for 20 min. Following centrifugation at 2,000×*g* for 5 min, approximately 1 ml of the supernatant was transferred to a new 2-ml tube and subsequently rinsed 3 times with 0.2 ml of sterile saline. After discarding the upper phase, the organic layer (50 to 100 μl) from the interface was retained for the subsequent experiment. The organic phase was subjected to 3 washes using 0.2 ml of sterile saline. To ensure purity, the interface was then rinsed with 0.2 ml of a 1:1 (v/v) methanol/water solution. Following centrifugation at 2,000×*g* for 5 min, the upper aqueous phase was discarded and the lower lipid-containing layer was collected. Methanol was added to the lower layer of lipid to adjust volume to 500 μl and dried overnight in a fume hood. The dried lipids were reconstituted with 50 μl of Triton X-100/isopropanol (v/v, 1:9). TG was determined with a triglyceride assay kit (Nanjing Jiancheng, China) in strict adherence to the manufacturer’s recommendations. RIPA lysis buffer was used to lyse equal amounts of tissue or cells, and the bicinchoninic acid technique was used to measure the protein content. The absolute amount of TG calculated was normalized to the protein concentration. Cellular or liver ATP levels were measured via a CheKine Micro ATP Content Assay Kit (Abbkine, China). The absolute amount of ATP calculated was normalized to the protein concentration, and then converted into a percentage of the control group.

### Liver and cellular NEFA-level assay

NEFA concentrations were measured using a commercial kit (Nanjing Jiancheng, China). Liver tissues or cells were homogenized in saline at a weight-to-volume ratio of 1:9 (g/ml) and subsequently centrifuged at 2,500×*g* for 10 min. The supernatant was collected and analyzed according to the assay kit instructions. The absorbance of each specimen was determined at 546 nm via a SpectraMax i3 plate reader (Molecular Devices, USA).

### Statistical analysis

All experiments were conducted with a minimum of 3 biological replicates, and the data are presented as mean ± standard deviation (SD). Statistical analyses were performed using Student’s *t* test for comparisons between 2 groups and one-way analysis of variance for comparisons among multiple groups with GraphPad Prism 8. A *P* value of less than 0.05 was considered statistically significant.

## Ethical Approval

The study involved human sample collection and use was approved by the Medical Ethics Committee of the Affiliated Hospital of Qingdao University (approval no. QYFY WZLL 30204) and complied with the Declaration of Helsinki. Each participant signed a written informed consent form. The animal experiments were conducted in compliance with ethical standards and received approval from the Ethics Committee of Medical College of Qingdao University (approval no. QDU-AEC-2024786 and QDU-AEC-2024767), adhering to institutional guidelines and regulations.

## Data Availability

All data generated or analyzed during this study are available from the corresponding author on reasonable request.
