# Supplementary material for: EVA1A Regulates Hepatic Lipid Homeostasis by Modulating CD36 Expression and Its Palmitoylation
Source: Research (Wash D C). 2025 Nov 25;8:1001. doi: 10.34133/research.1001 (PMC12645495; doi:10.34133/research.1001)
Supplement: Supplementary 1 — Tables S1 to S5 Figs. S1 to S13 [file research.1001.f1.docx]

**EVA1A Regulates Hepatic Lipid Homeostasis by Modulating CD36 Expression and Its Palmitoylation**

**Short Title：EVA1A Regulates Hepatic Lipid Homeostasis via CD36**

Di Yang ^1^§, Lianhui Li ^1^§, Kailai Zang ^1^§, Wanyong Ma ^2^, Yuling Yang ^3^, Yani Sun^1^, Bingqiang Zhang ^4,5^, Zunshuang Gong ^1^, Mingkang Yu ^1^, Qiyuan Du ^1^, Xiaokun Liu ^1^, Zhe Wang ^1^, Qiyue Xu ^6^, Ning Li ^1^*

^1^ Department of Biochemistry and Molecular Biology, School of Basic Medicine, Qingdao Medical College, Qingdao University, Qingdao, China

^2^ Emergency department, Yantai Affiliated Hospital of Binzhou Medical University, Yantai, China

^3^ Department of infectious diseases, The Affiliated Hospital of Qingdao University, Qingdao University, Qingdao, China

^4^ Qingdao Restore Biotechnology Co., Ltd., Qingdao, China

^5^ Qingdao Engineering Research Center for Cellular Immunity and Early Cancer Screening, Qingdao, China

^6^ Department of business, Qingdao University of Technology, Qingdao, China

§ These authors contributed equally to this work.

* Address correspondence to: [lining@qdu.edu.cn](mailto:lining@qdu.edu.cn) ORCID: 0000-0002-1895-4983

**Supplementary figures and legends**

**
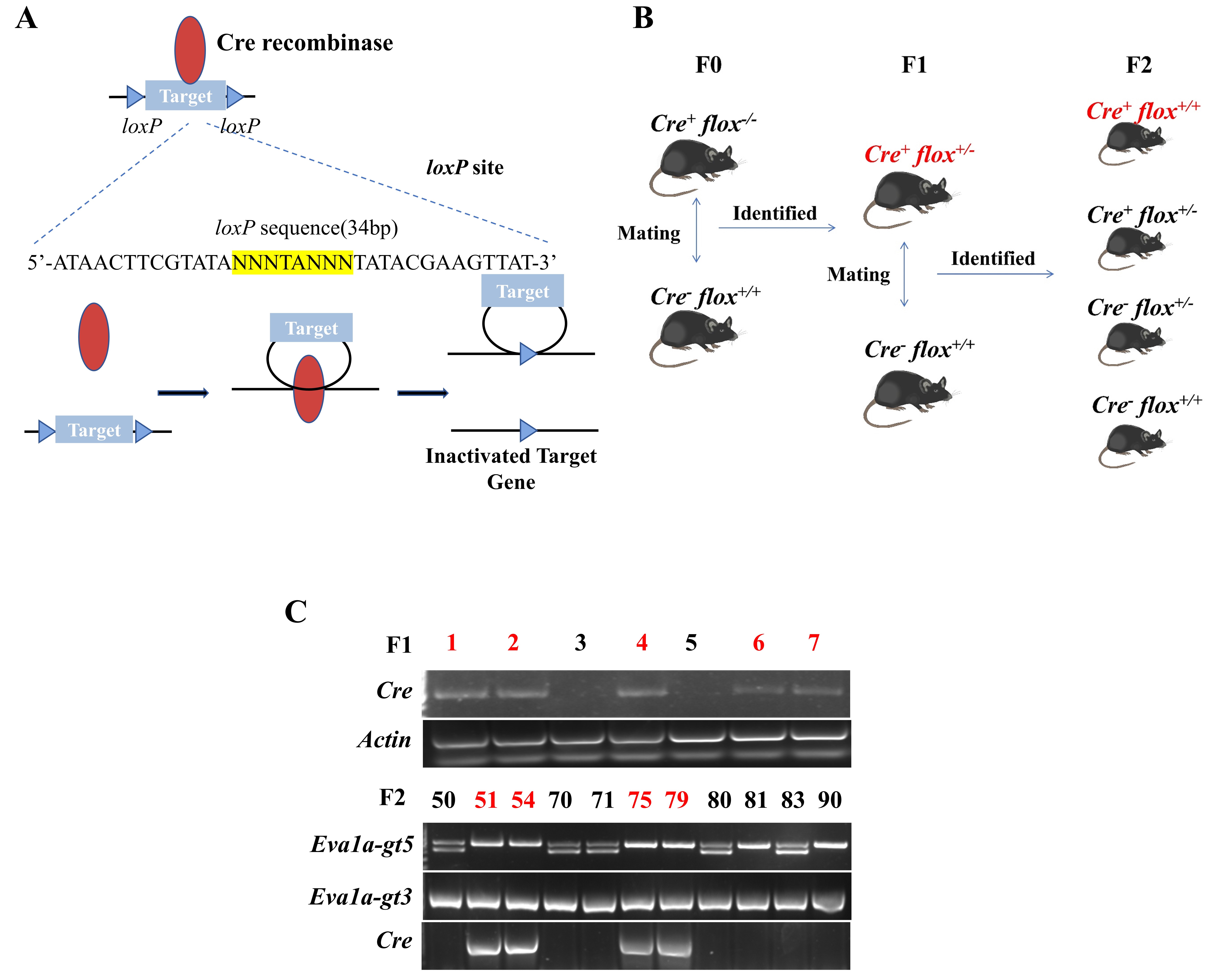
**

**Fig. S1.** **Liver specific *Eva1a* gene knockout mouse construction and identification.**

(**A**) The schematic diagram of Cre-loxp system. Two loxp sites were inserted on either side of the third exon of EVA1A(34-156aa). (**B**) Reproductive strategies of liver specific *Eva1a* knockout mice. Alb-Cre mice were crossed with *Eva1a^flox/flox^* mice to obtain the F1 generation. *Cre*^+^ mice in the F1 generation were identified by mouse tail PCR, then crossed with *Eva1a^flox/flox^* mice to obtain the F2 generation. In the F2 generation, *Cre*^+^*Eva1a^f^**^lox+/+^* mice were the liver-specific *Eva1a* knockout group (*Eva1a^-/-^*), *Cre*^-^ *Eva1a^flox+/+^* mice in the same litter served as the control group (*Eva1a^+/+^*). (**C**) The tail of mice was clipped at 6 weeks of age and extracted DNA for detection *Cre* gene by PCR in F1 generation, using the primer of *Cre* gene. In F2 generation, *Cre* gene and two loxp sites were detected by PCR. *Eva1a-gt5* primers were used to amplify the left loxp sequence, *Eva1a-gt3* primers were used to amplify the right loxp sequence. After the band was amplified with *Eva1a-gt3* as primer, only the upper band was amplified with *Eva1a-*gt5 primer, indicating the *Eva1a ^flox+/+^* genotype. If two bands were amplified with *Eva1a-gt5* primer, it indicates the *Eva1a^flox+/-^*genotype*.*

**
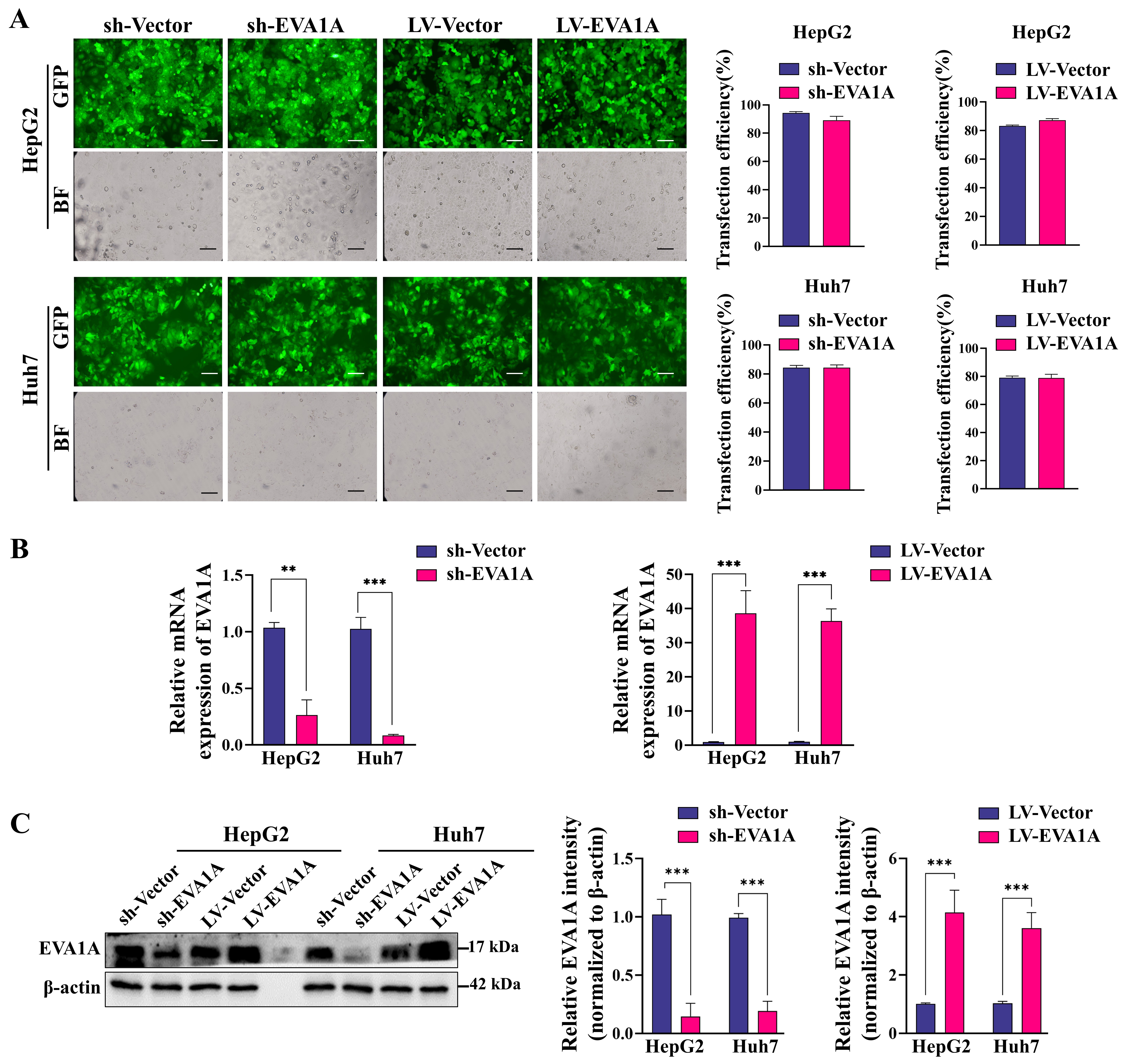
**

**Fig. S2. Identification of EVA1A stable knockdown and stable overexpression cell lines.**

(**A**) The infection efficiency of LV-EVA1A-GFP and the transfection efficiency of sh-EVA1A-GFP were determined by fluorescence microscopy imaging. Scale bars: 50 μm. Transfection efficiency was quantified in the right panels. (**B**) RT-qPCR analysis of EVA1A expression in HepG2 and Huh7 cells stably transfected with GFP-labelled sh-EVA1A and LV-EVA1A. (**C**) Western blot analysis of EVA1A expression in HepG2 and Huh7 cells stably transfected with GFP-labelled shEVA1A and LV-EVA1A. Protein levels were quantified with Image J in the right panels. The data are shown as the means ± SDs from three independent experiments. ***P* < 0.01, ****P* < 0.001. BF: bright field.


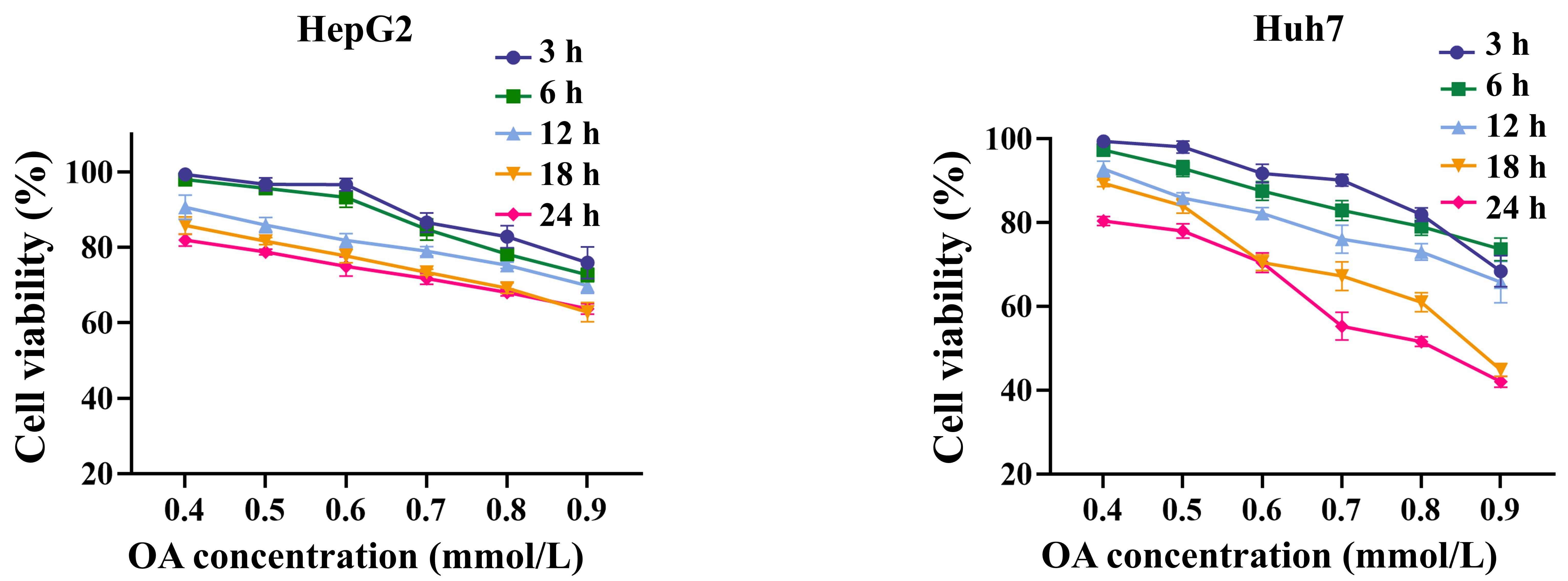


**Fig. S3.** **The effect of oleic acid on cell viability.** HepG2 and Huh7 cells were treated with different concentrations of oleic acid (OA) for indicated time. Cell viability was analyzed by CCK-8 assay.


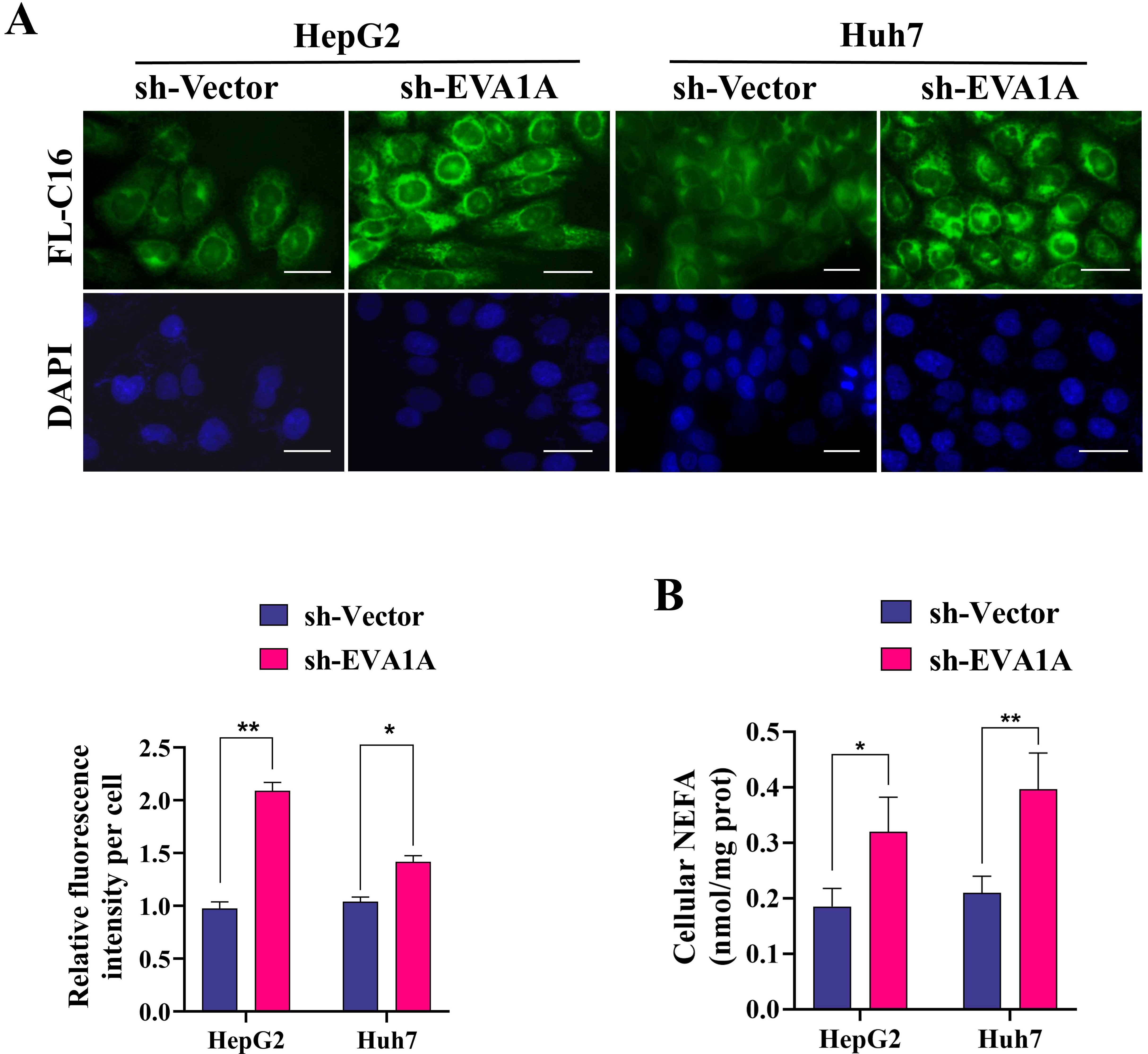


**Fig. S4. EVA1A** **overexpression inhibits** **fatty acids uptake in HepG2 and Huh7 cells.**

(**A**) Representative fluorescence images of cellular fatty acids (stained with BODIPY FL-C16, green) and nuclei (stained with DAPI, blue) in control cells or EVA1A-knockdown HepG2 and Huh7 cells. Scale bars: 20 µm. The fluorescence signal intensity of FL-C16 per cell was quantified by Image J in the lower panels. Values were means ± SDs, *n* = 50 cells from three independent experiments. (**B**) The cellular NEFA levels in control cells or EVA1A-knockdown HepG2 and Huh7 cells. The data are shown as the means ± SDs from three independent experiments. **P* < 0.05, ***P* < 0.01





**Fig. S5.** **EVA1A negatively regulates the lipid droplet accumulation in HepG2 and Huh7 cells.**

(**A**) Representative microscopy images of Bodipy493/503 staining for lipid droplets in control cells or EVA1A-knockdown HepG2 and Huh7 cells untreated or treated with 400 µM OA for 6 h or 12 h. Scale bars: 5 µm. (**B**) Representative fluorescence microscopy images of Bodipy493/503 staining for lipid droplets in control cells or EVA1A overexpressed HepG2 and Huh7 cells untreated or treated with 400 µM OA for 6 h or 12 h. Scale bars: 5 µm. The fluorescence signal intensity of Bodipy493/503 per cell was quantified by Image J in the lower panels (*n* = 50). All the data are shown as the means ± SDs from three independent experiments. **P* < 0.05, ***P* < 0.01


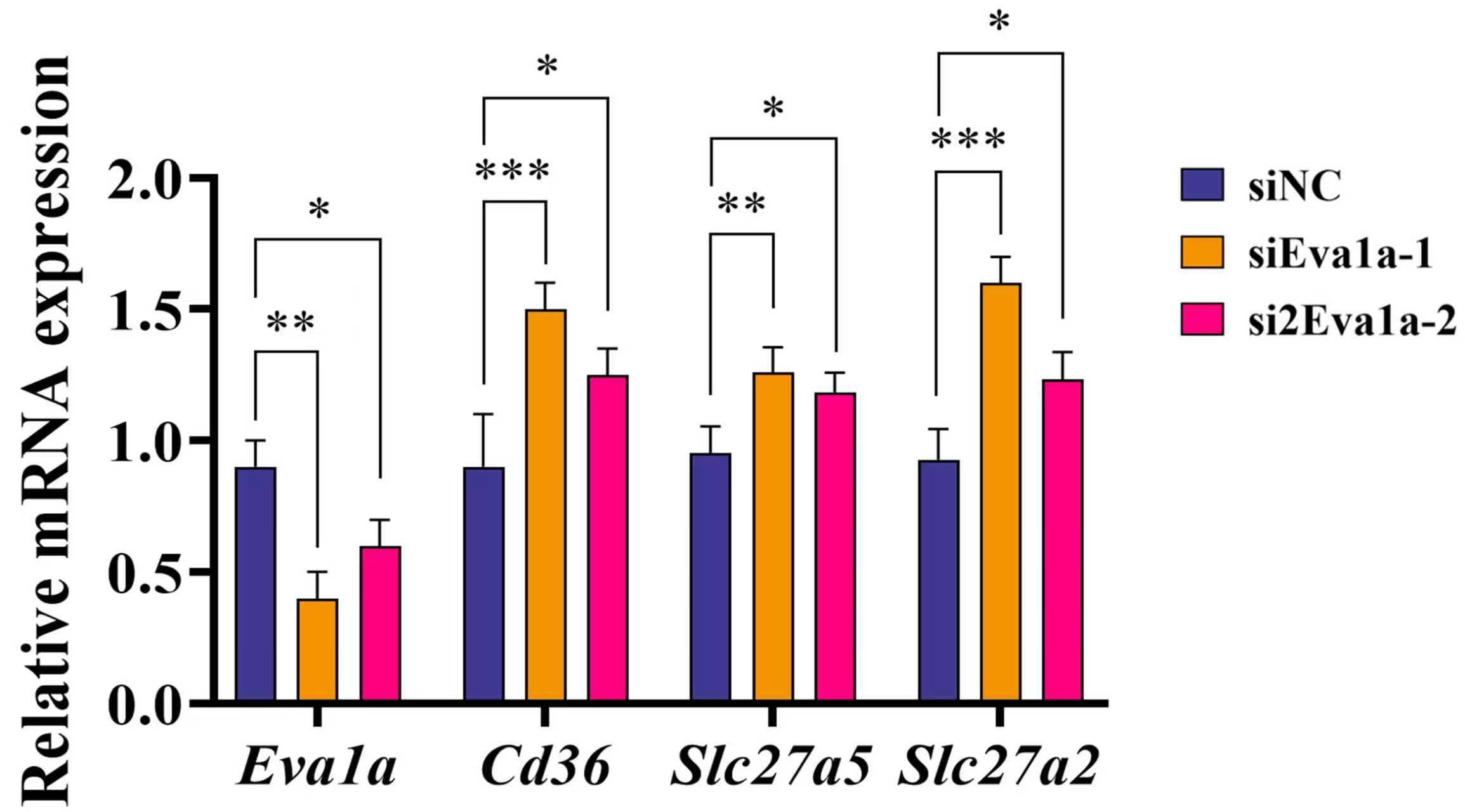


**Fig. S6. *Eva1a* knockdown enhances the expression of genes associated with fatty acid uptake in primary rat hepatocyte.** The primary rat hepatocytes were transfected with siNC or siEva1a-1 or siEva1a-2, 48 h later, RT-qPCR analysis of the relative mRNA levels of *Eva1a* and genes associated with fatty acid intake (*Cd36*, *Slc27a5* and *Slc27a2*). The data are shown as the means ± SDs from three independent experiments. **P* < 0.05, ***P* < 0.01, ****P* < 0.001.





**Fig. S7.** **CD36 knockdown blocks fatty acid uptake induced by EVA1A deletion.**

(**A**) The knock-down efficiency of CD36 was determined by RT-qPCR. (**B**) Representative fluorescence images of BODIPY FL-C16 staining for fatty acids in cells treated as (**A**). Scale bars: 20 µm. The fluorescence signal intensity of FL-C16 per cell was quantified by Image J in the lower panels (*n* = 50). (**C**) Cellular NEFA levels in cells treated as (**A**) indicated. All data are shown as the means ± SDs from three independent experiments. **P* < 0.05, ***P* < 0.01, ****P* < 0.001


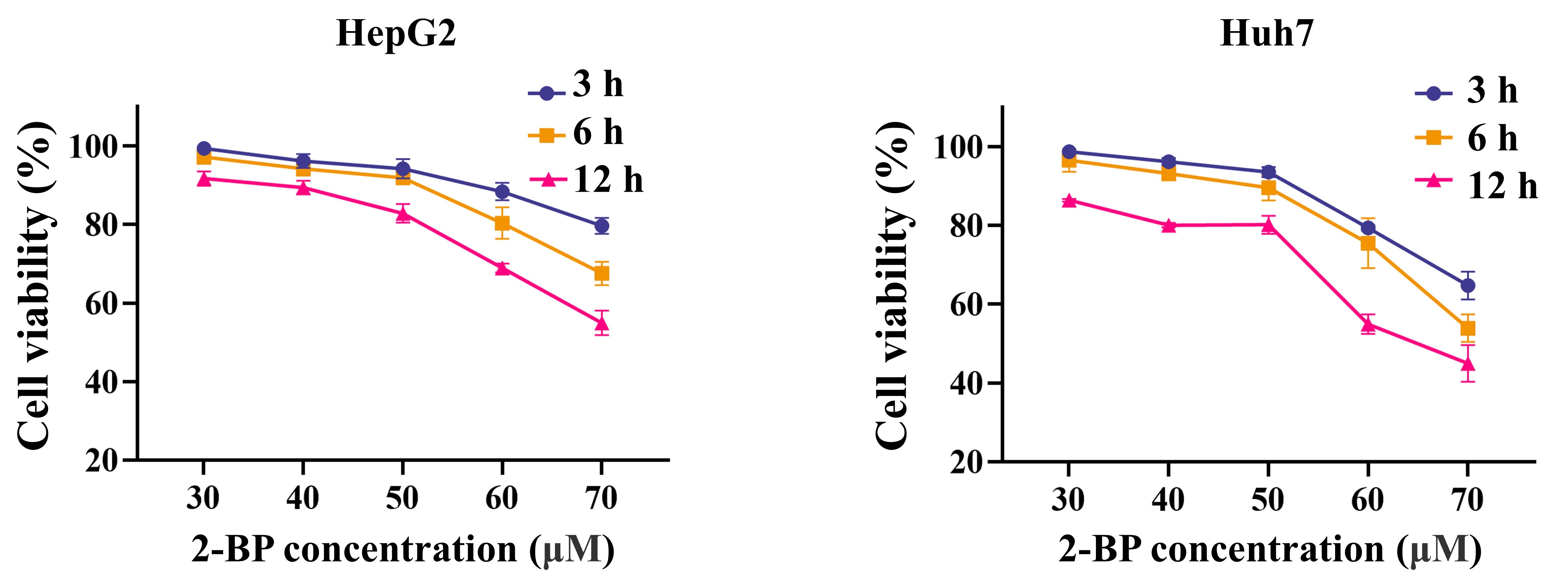


**Fig. S8. The effect of 2-Bromohexadecanoic acid (****2-BP) on cell viability.** HepG2 and Huh7 cells were treated with different concentrations of 2-BP for indicated time. Cell viability was analyzed by CCK-8 assay.





**Fig. S9. Inhibition of CD36** **palmitoylation diminishes EVA1A deficiency-induced fatty acid uptake.**

(**A**) Western blot analysis of CD36 expression in EVA1A-knockdown HepG2 and Huh7 cells transfected with empty (pcDNA3.1) vector or Flag-tagged CD36 wild type (CD36-wt) or CD36 mutant (CD36-mut) plasmid. Protein levels were quantified in the right panels. (**B**) Representative fluorescence images of BODIPY FL-C16 staining for fatty acids in cells treated as (**A**). Scale bars: 10 µm. The fluorescence signal intensity of FL-C16 per cell was quantified by Image J in the right panels (*n* = 50). (**C**) Cellular NEFA levels in cells treated as (**A**) indicated. (**D**) The EVA1A-knockdown cells were treated with 50 µM 2-BP for 6 h, then were subjected to BODIPY FL-C16 staining. Scale bars: 10 µm. (**E**) Cellular NEFA levels in cells treated as (**D**) indicated. All the data are shown as the means ± SDs from three independent experiments. **P* < 0.05, ***P* < 0.01, ****P* < 0.001


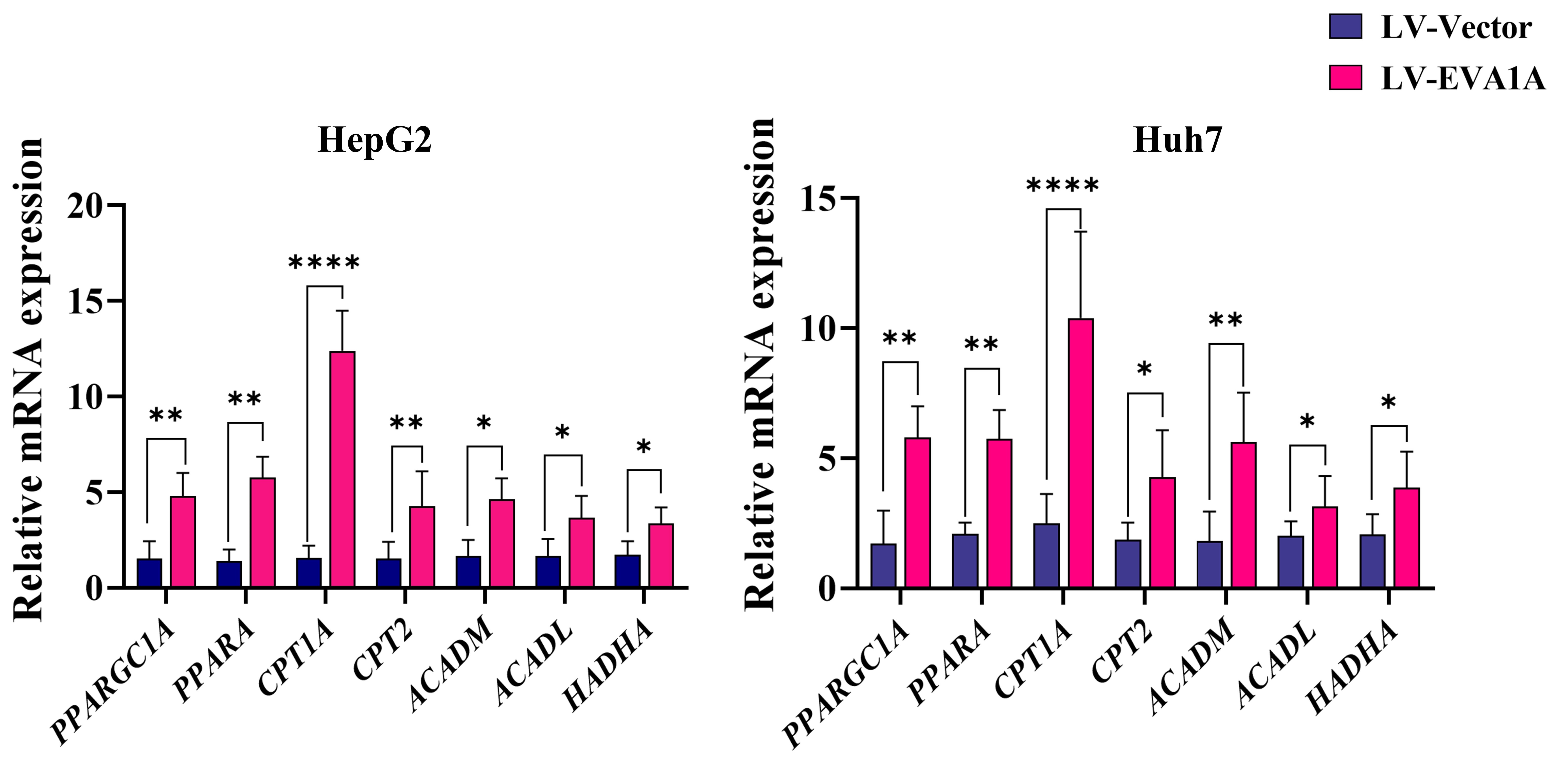


**Fig. S10.** **The impact of EVA1A overexpression on the expression of genes** **associated with fatty acid β-oxidation.**

RT-qPCR analysis of genes related to fatty acid β-oxidation and their corresponding transcription factors in control cells or EVA1A-overexpressing HepG2 and Huh7 cells.


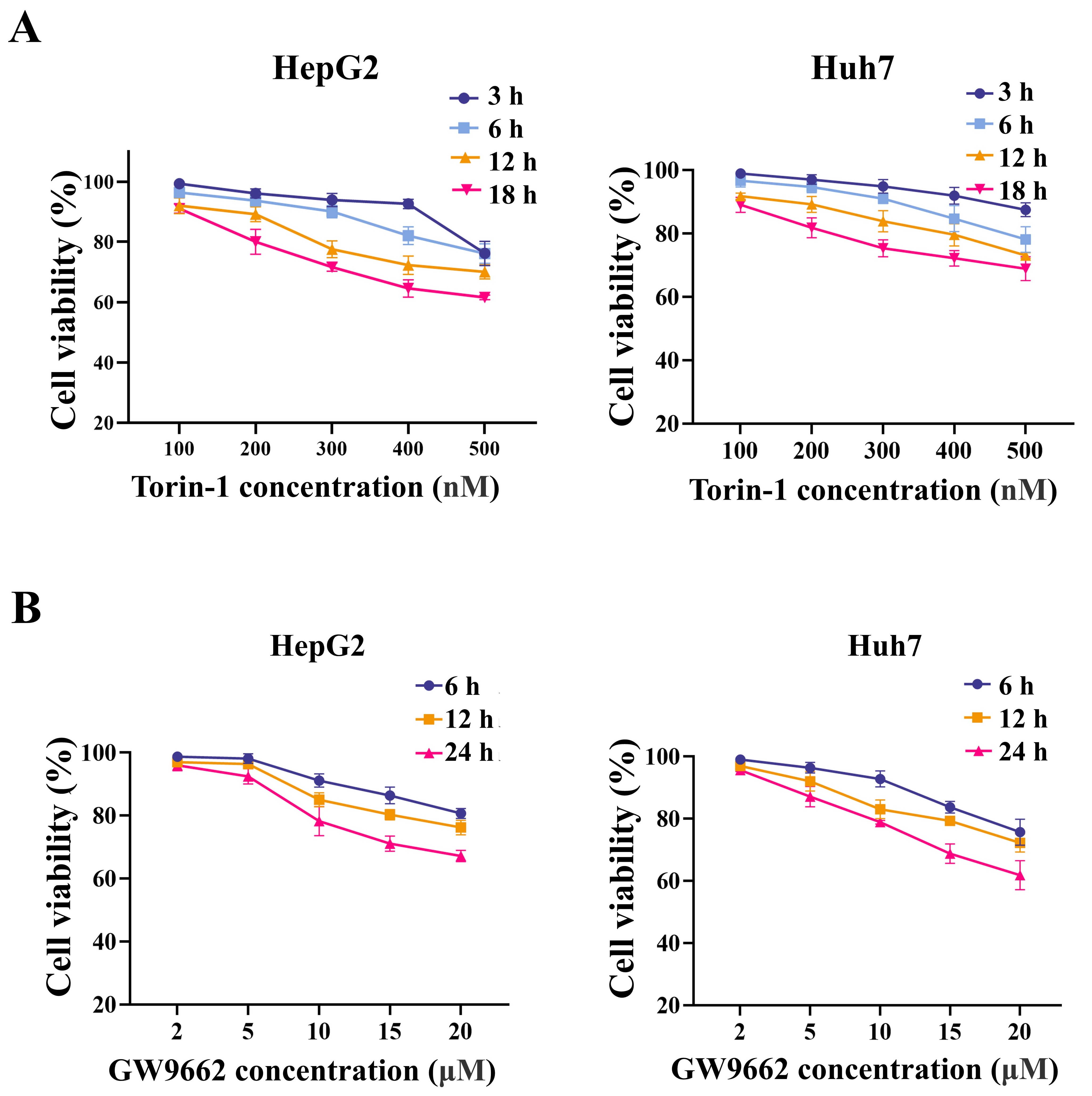


**Fig. S11.** **The effects of Torin-1 and GW9662 on cell viability.**

(**A, B**) HepG2 and Huh7 cells were treated with different concentrations of Torin-1(**A**) or GW9662(**B**) for indicated time. Cell viability was analyzed by CCK-8 assay.


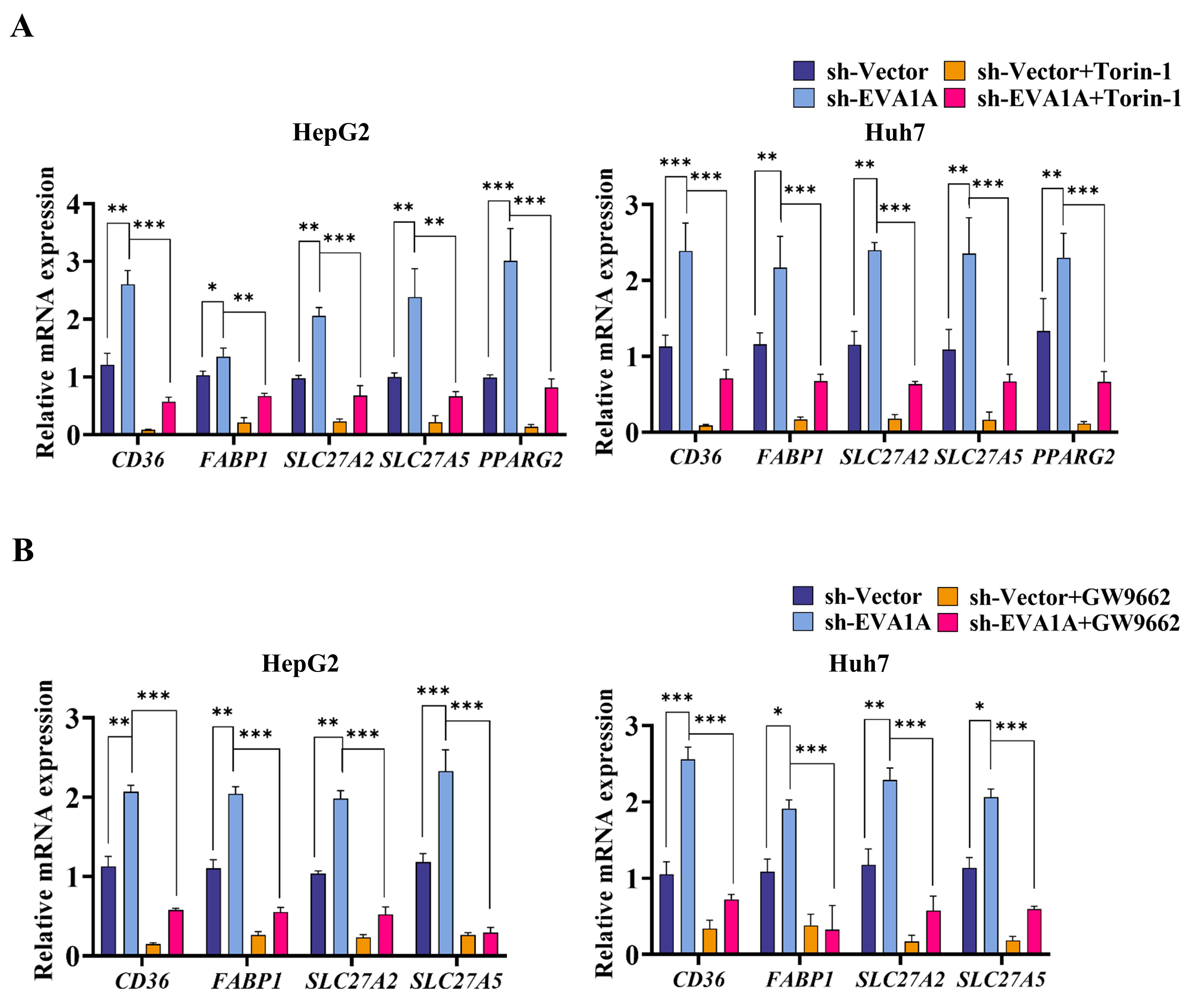


**Fig. S12. The effects of Torin-1 and GW9662 on the expression of genes associated with fatty acid transport and PPARγ2.**

(**A, B**) Relative mRNA levels of genes related to fatty acid transport and PPARγ2 in control or EVA1A-knockdown HepG2 or Huh7 cells with or without Torin1 (300 nM, 6 h) (**A**) or GW9662 (5 µM, 12 h) (**B)** treatment. All the values are shown as the means ± SDs from three independent experiments. **P* < 0.05, ***P* < 0.01, ****P* < 0.001

**
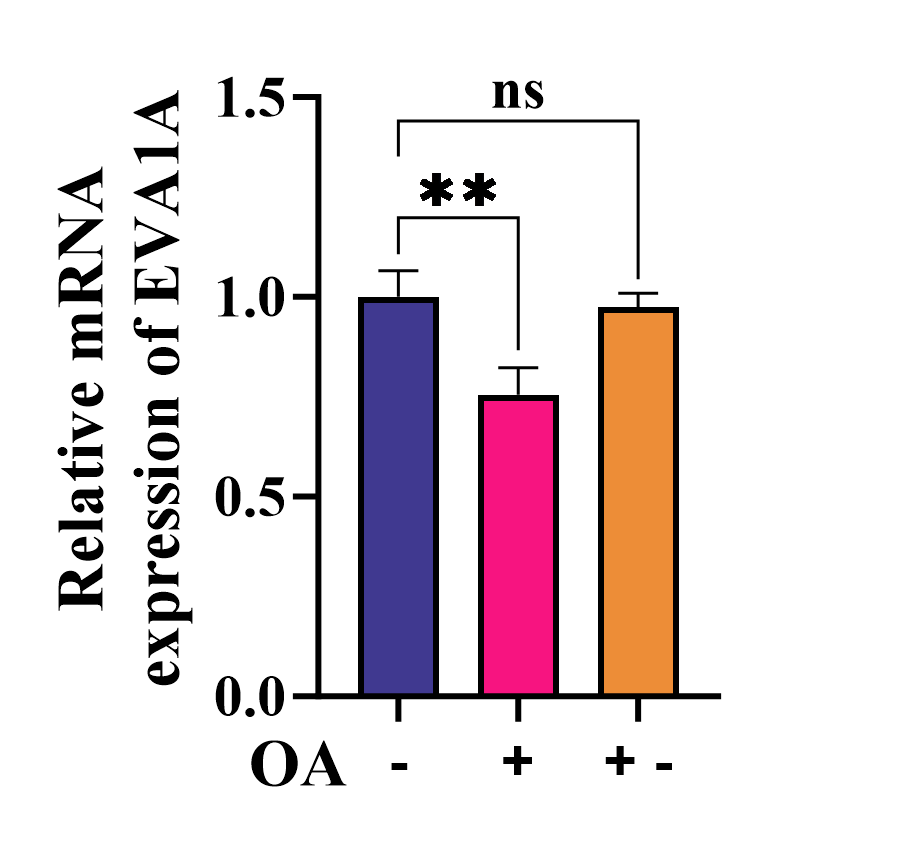

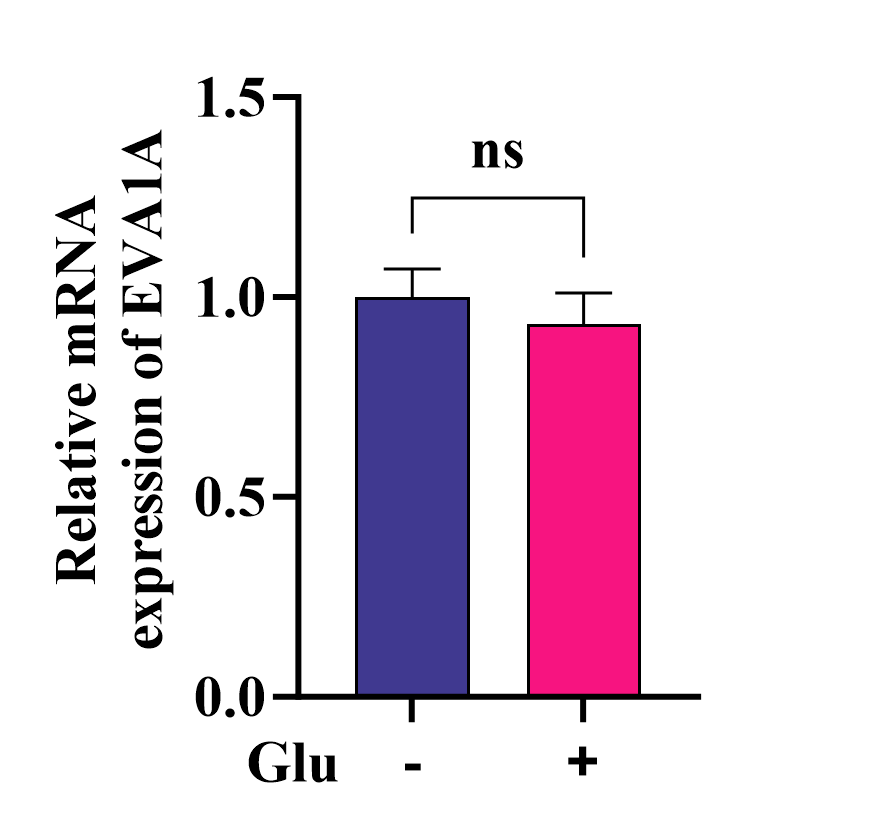
A B**

**Fig. S13. The effects of oleic acid or glucose stimulation on the expression of EVA1A.**

(A) RT-qPCR analysis of relative EVA1A mRNA levels in HepG2 cells untreated, treated with OA (400 µM, 12 h), or subjected to OA withdrawal for 12 h after treatment. (B) Relative EVA1A mRNA levels in HepG2 cells with or without glucose (30 mM, 12 h) treatment.

**Supplementary Materials**

**Supplemental Table S1**

Primers for mouse tail gene identification

| **Gene names** | **sequences** |
| --- | --- |
| Mouse *Eva1a-gt3* | forward: 5'-TCTGAGGCGGAAAGAACCAG-3'  reverse: 5'-CAGCCCAGGAAATAGGATGA-3' |
| Mouse *Eva1a-gt5* | forward: 5'-ATCTGTTAGGGACAAGGGTA-3'  reverse: 5'-CAAAGGAGAATFFCAAATGG-3' |
| Mouse *Alb Cre* | forward: 5'-TGGCAAACATACGCAAGG-3'  reverse: 5'-CGGCAAACGGACAGAAGCA-3' |

**Supplemental Table S2**

The primer sequences for RT-qPCR analysis of genes in mice liver tissues

| **Gene names** | **sequences** |
| --- | --- |
| *Fasn* | forward: 5'-GGCACACACAATGGACCCCCAG-3'  reverse: 5'-CCCAGACGCCAGTGTTCGTTCC-3' |
| *Acly* | forward: 5'-GTGTCCTCGGCCTCCTCTGGTT-3'  reverse: 5'-TTCCCAGCCCGAGCACAGATGA-3' |
| *Pnpla2* | forward: 5'-TCCAAGGGGTGCGCTATGTGGA-3'  reverse: 5'-GTGGAGCTGTCCTGAGGGCAGA-3' |
| *Cd36* | forward: 5'-TGACGTGGCAAAGAACAGCAGC-3'  reverse: 5'-AGACACAGTGTGGTCCTCGGGG-3' |
| *Mttp* | forward: 5'-CAGCACCTCCGGACTTCGAGGA-3'  reverse: 5'-TGAGCAGAGGTGACGGCATCCA-3' |
| *Dgat2* | forward: 5'- GGAGGTGCAGCTGAGTCCCTGA-3'  reverse: 5'- CAGATCAGCTCCATGGCGCAGG-3' |
| *Agpat1* | forward: 5'-GCAGCCCTACGTGGTTGTGTCC-3'  reverse: 5'-GCTTGGCAATGGGCACACAACG-3' |
| *Mgat1* | forward: 5'-AAGTTCAAGTTCCCGGCCGCTG -3'  reverse: 5'-TCTCAGCAGTGGGTAGGTGGCC-3' |
| *Cpt1a* | forward: 5'-ACACCACTGGCCGCATGTCAAG-3'  reverse: 5'-AGGAGAGCAGCACCTTCAGCGA-3' |
| *Cpt2* | forward: 5'-CAAACACGGCCGCACAGAGACT-3'  reverse: 5'-CTCGCCCACACTGTGCTTGGAG-3' |
| *Fabp1* | forward: 5'-GCAATAGGTCTGCCCGAGGACC-3'  reverse: 5'-CCCCAGGGTGAACTCATTGCGG-3' |
| *Slc27a2* | forward: 5’-CTGCGGGCCTTCCTGGAACAAG-3’  reverse: 5’-CCCCTGTCGTAGGCCCAGTTGA-3’ |
| *Slc27a5* | forward: 5’-CAACACTGGGGACGTGCTGACC-3’  reverse: 5’-CGTTTTCGCCCTTCCACCGGAA-3’ |
| *Acaca* | forward: 5’-TCGGATCGGTTCCTTTGGGCCT-3’  reverse: 5’-TGTTCGCTGCCACGTAGATGCG-3’ |
| *Apob* | forward: 5’-AAAGCTGCTCTCCAGGCCCTGA-3’  reverse: 5’-TCTCCACGGGAGCGACACCATT-3’ |
| *Lipe* | forward: 5’-GGGCTTCCAGTTCACACCTGCC-3’  reverse: 5’-GAACTGGCGGTCACACTGAGGC-3’ |
| *Lypla1* | forward: 5’-GGCAACAACATGTCCGCTCCGA-3’  reverse: 5’-GCTTCTGCCCATCCATGCCCTG-3’ |
| Pparg2 | forward: 5’-ATGGGTGAAACTCTGGGAGATT-3’  reverse: 5’-CGAAGTTGGTGGGCCAGAATGG-3’ |
| *Zdhhc4* | forward: 5’-CACCCTGACTTGTGCCGCCAAT-3’  reverse: 5’-AAATCGCAAGTGGGGCACCTCG-3’ |
| *Zdhhc5* | forward: 5’-CCGAGAACCCTCACCAGTCCGT-3’  reverse: 5’-CTTCCTCACGGCCTGCAAGTGG-3’ |
| *Srebf1* | forward: 5’-ACGTTTCTTCCTGAGCAGCGCC-3’  reverse: 5’-GACCTACAGGGTGGCAGAGCCA-3’ |
| *Scd1* | forward: 5’- CTCTGGTGCTCAACGCCACCTG-3’  reverse: 5’- TAGTTGTGGAAGCCCTCGCCCA -3’ |
| *Acadm* | forward: 5’-CCAGAGAGCAGCCTGGGAGGTT-3’  reverse: 5’-CCTCCGAAAATCTGCACGGCGT-3’ |
| *Acadl* | forward: 5’-ACGTCTGGACTCCGGTTCTGCT-3’  reverse: 5’-ACTCCCACATGTACCCCCAGCC-3’ |
| *Hadha* | forward: 5’-GGGCTTGGCTTTCCCCCTTGTC-3’  reverse: 5’-AGGAGCAGCTGGCATGGGGTAA-3’ |
| *Eva1a* | forward: 5’- CCTTGGCCGCCTTGGTGATGAG-3’  reverse: 5’- TACCATCCTCGCTGTCGCTGCT-3’ |
| *Actb* | forward: 5’- CCCGGGCTGTATTCCCCTCCAT-3’  reverse: 5’- CCTCTCTTGCTCTGGGCCTCGT-3’ |

**Supplemental Table S3**

The primer sequences for RT-qPCR analysis of genes in primary rat hepatocytes

| **Gene names** | **sequences** |
| --- | --- |
| *Cd36* | forward: 5'- ACGACTGCAGGTCAACATAC-3'  reverse: 5'- CGATGGTCCCAGTCTCATTTAG-3' |
| *Slc27a2* | forward: 5'- GCTGACATTGTGGGACTGGT-3'  reverse: 5'- CGATGCGACCTTCATGACCT-3' |
| *Slc27a5* | forward: 5'- AAGCTCTACCAGCATGTCCG-3'  reverse: 5'- TTTCGGCCTTGTTGTCCAGT-3' |
| *Eva1a* | forward: 5'- AGCCTGGGCCTATATCTCAGAA-3'  reverse: 5'- CACTACCATCCTCGCTGTCG-3' |
| *Actb* | forward: 5’- AACACAGTGCTGTCTGGTG-3’  reverse: 5’- GTAACAGTCCGCCTAGAAGC-3’ |

**Supplemental Table S4**

The primer sequences for RT-qPCR analysis of genes in human liver tissues or human cells

| **Gene names** | **Oligonucleotide sequences** |
| --- | --- |
| *CD36* | forward: 5’- CAGGTCAACCTATTGGTCAAGCC-3’  reverse: 5’- GCCTTCTCATCACCAATGGTCC -3’ |
| *ZDHHC4* | forward: 5’-AGGCTTGGGGGACTCAGGCATT-3’  reverse: 5’-ATCTGGCTTGCCAAAACGGGGG-3’ |
| *ZDHHC5* | forward: 5’- AGGCTTGGGGGACTCAGGCATT -3’  reverse: 5’- ATCTGGCTTGCCAAAACGGGGG -3’ |
| *PPARG2* | forward: 5’- ATGGGTGAAACTCTGGGAGATT-3’  reverse: 5’- CAAAGTTGGTGGGCCAGAATGG-3’ |
| *LYPLA1* | forward: 5’-TAACATGTCAACCCCGCTGCCC-3’  reverse: 5’-TTCTGCCCATCCGTGCCCAGTA-3’ |
| *FABP1* | forward: 5’-GGAGGAATGTGAGCTGGAGACA-3’  reverse: 5’-TATGTCGCCGTTGAGTTCGGTC-3’ |
| *SLC27A2* | forward: 5'- GTGGAGAAAGATGAACCTGTCCG-3'  reverse: 5'-CTGAGCCTTTGCTCCAGCATAG -3' |
| *SLC27A5* | forward: 5'-TCTGGGATGACTGTCGGCAGCA-3'  reverse: 5'-TCAGCCCGTAGTCCATTGCCCA-3' |
| *CPT1A* | forward: 5'-AAATGTCGCACGAGCCCAGACG-3'  reverse: 5'-TCCGTCCTCCCCTCTCGGAAGA-3' |
| *CPT2* | forward: 5'-ATGGGGATGGCACAAACCGCTG-3'  reverse: 5'-GAGCACTGCCACACCATCACCC-3' |
| *PPARGC1A* | forward: 5'-ACTCCACCAAGAAAGGGCCGGA-3'  reverse: 5'-GGGCCGCTTGGTCTTCCTTTCC-3' |
| *PPARA* | forward: 5'-ACGCTTTCACCAGCTTCGAGCC-3'  reverse: 5'-AGGCCTTGTCCCCGCAGATTCT-3' |
| *ACADM* | forward: 5'-ACCAGAGAGCAGCTTGGGAGGT-3'  reverse: 5'-TGCCTCCAAGTATCTGCACAGCA-3' |
| *ACADL* | forward: 5'-GTTTGGACTCCGCCACTGCTTG-3'  reverse: 5'-GGCTGAACTCTGGCATCCACAT-3' |
| *HADHA* | forward: 5'-GCCGACATGGTGATTGAAGCTG-3'  reverse: 5'-GGAGAGCAGATGTGTTACTGGC-3' |
| *EVA1A* | forward: 5'-AGATGGCTTTGCTCAGCAACA-3'  reverse: 5'-GATGCACACGCCAGAAACAA-3' |
| *ACTB* | forward: 5'- AGGATTCCTATGTGGGCGAC -3'  reverse: 5'- ATAGCACAGCCTGGATAGCAA -3' |

**Supplemental Table S5**

List of antibodies used in Western blot or immunofluorescence.

| **Antibody** | **Source** | **Catalog Number** |
| --- | --- | --- |
| anti-CD36 | ABclonal | A19016 |
| anti-CD36 | BOSTER | MA01189 |
| anti-CD36 | Proteintech | 66395-1-Ig |
| Alexa-Fluor®647-anti-CD36 | Abcam | ab311164 |
| anti-EVA1A | ABclonal | A8070 |
| anti-FATP2 | Abmart | TD6222 |
| anti-FATP5 | Abmart | TD3845 |
| anti-ZDHHC4 | Abmart | PHB0612 |
| anti-ZDHHC5 | Abmart | PH1196 |
| anti-APT1 | Abmart | PA3382 |
| anti-Tom20 | Proteintech | 11802-1-AP |
| anti-VDAC | Proteintech | 10866-1-AP |
| anti-ATP1A | Proteintech | 14418-1-AP |
| anti-PPARγ2 | Santa Cruz | sc-166731 |
| anti-mTOR | Proteintech | 66888-2-Ig |
| anti-p-mTOR | Proteintech | 14485-1-AP |
| anti-p70S6K | Proteintech | 14485-1-AP |
| anti-p-p70S6K | ABclonal | AP0502 |
| anti-CPT1A | Proteintech | 15184-1-AP |
| anti-β-actin | ABclonal | AC026 |
| anti-GAPDH | ABclonal | A19056 |
